# Field evaluation of PGP *Bacillus* sp. strain D5 native to *Crocus sativus,* in traditional and non traditional areas, and mining of PGP genes from its genome

**DOI:** 10.1038/s41598-021-84585-z

**Published:** 2021-03-09

**Authors:** Shanu Magotra, Nancy Bhagat, Sheetal Ambardar, Tahir Ali, Barbara Reinhold Hurek, Thomas Hurek, Praveen Kumar Verma, Jyoti Vakhlu

**Affiliations:** 1 Metagenomic Laboratory, School of Biotechnology, University of Jammu, Jammu, 180006 India; 2 National Center for Biological Sciences, Bellary Road, Bangalore, 560065 India; 3 Department of Microbe-Plant Interactions, Faculty of Biology and Chemistry, University of Bremen, P.O. Box 33 04 40, Bremen, Germany; 4 Plant Immunity Laboratory, National Institute of Plant Genome Research (NIPGR), New Delhi, 110067 India; 5 University Institute of Biotechnology, Chandigarh University, Punjab, 140413 India

**Keywords:** Biotechnology, Microbiology, Molecular biology

## Abstract

Native *Bacillus* sp. strain D5 coded as (Bar D5) has been isolated from the saffron corm that showed plant growth promotion (PGP) properties and also inhibits the growth of corm rot causing *Fusarium oxysporum* R1 (Fox R1) in-vitro. Bar D5 was more efficient PGP bacterium in comparison to earlier reported native bio-formulations by our group. Pot assays and field evaluation of Bar D5 confirmed its in-vivo efficacy for PGP traits and biocontrol activity as well. Pot trials were followed by field trials at traditional (Kishtwar) and non-traditional (R.S Pura) saffron cultivation areas in Jammu and Kashmir. At both places, Bar D5 bio-formulation treatment led to the increase in root number & length, shoot number & length, flower number and number & weight of daughter corms. Additionally, it also decreased the corm rot disease incidence significantly. Priming of corms with bio-formulation resulted in the reduction of pathogenic fungal load by three fold at the depth of corm sowing from ground level. The shelf life/viability of Bar D5 based bio-formulation was found to be 52% (viable spores) for one year at room temperature. Draft genome sequence of Bar D5 revealed the presence of genes necessary for PGP and biocontrol activity. Further, confirmation of gene sequences and annotation was done by amplification, re-sequencing and mapping of PGP and biocontrol genes on draft genome. Bar D5 based bio-formulation can be provided to companies/researchers interested in saffron cultivation or bio-formulation production for commercial exploitation, since saffron is grown as revenue crop across continents. The present study bridges the gap between genomics and its field application.

## Introduction

*Crocus sativus,* native to Mediterranean and Western Asia, is cultivated for its highly valuable stigma. This costliest spice in the world is produced majorly in Iran, Spain, Morocco and India^1^. Due to its medicinal, cosmetic, culinary uses, laborious collection and cultivation process, one pound of dried stigma costs around 70 K INR^2^. It is a perennial herb with male sterile flowers and propagates asexually via modified underground stem, corm. Corm quality, size and density have a significant effect on the number of daughter corms, flower production and on overall yield^3–6^. Saffron cultivation in the state of Jammu and Kashmir in India is on decline. According to a survey conducted by the Jammu and Kashmir state agriculture department, there has been a major decline in saffron yield from 15 tons in 1997 to 9 tons in 2015^7^. Among various biotic factors, corm rot caused by *Fusarium oxysporum* is the most devastating^8–10^.

To increase the yield and to control the diseases, farmers are using chemical fungicides and fertilizers. The noxious these chemicals have on the environment as well as on humans are well established^11,12^. Excessive use of these chemicals also affects soil health and the profitable microflora, thereby putting selection pressure resulting in the evolution of resistant pathotypes^13^.

Plant associated bacteria have been found to be effective biological control agents, plant growth promoters and also find their importance in the suppressive soil phenomenon^14–16^. The use of specific microbes for the suppression of diseases and promotion of plant growth can potentially provide an alternative to chemical fertilizers & fungicides. *Pseudomonas* and *Bacillus* are amongst the most popular biocontrol agents used for the preparation of bio-formulations and have been extensively used commercially in agriculture^17–19^. Genus *Bacillus* is regarded as one of the classical genera for the biological control of plant diseases. The heat and desiccation-resistant spores make it more potent biological control agents. The ease of stable formulation, GRAS (generally regarded as safe) status by the US food and drug administration are additional properties that make *Bacillus* most used plant growth promoting microbe^20,21^. A number of species of *Bacillus* such as *B. pumilus, B. amyloliquefaciens, B. cereus, B. subtilis, B. licheniformis, B. megaterium, B. mycoides* and *B. Velezensis* have been commercialized as bio-formulation^22–25^.

Bacterial associations with saffron cormosphere have been explored and reported earlier by our group by cultivation dependent^26^ and cultivation independent metagenomics approach^27^. In our initial studies, all bacteria were evaluated for plant growth promoting properties but thereafter the emphasis was put on *Bacillus* (for above mentioned reasons) from fields, roots and corm of saffron^8,28^. Out of various PGPB isolated, characterized and reported by our group earlier, ***Bacillus*** **sp. strain D5** (Bar D5) has been now found to be the most promising PGPB, as it inhibits the growth of corm rot causing fungus *Fusarum oxysporum* R1 and promotes the growth & yield of saffron (in pots as well as in the fields). In the present study, we report field evaluation of Bar D5 as biocontrol-biofertilizer in traditional and non-traditional saffron cultivation areas. In addition, genome of Bar D5 was sequenced, annotated and the genes responsible for PGP activities, antifungal activity, and plant association were identified in draft genome of Bar D5. The technology for this bio-formulation is ready for commercialization.

## Materials and methods

### Sample collection

Corm samples were collected from saffron fields during three different stages of *Crocus sativus* life cycle i.e. vegetative stage (Mar–Apr), dormant stage (Jul–Aug) and flowering stage (Oct–Nov) in 2015 from Kishtwar district (33° 19′ 12.00ʺ N latitude and 75° 46′ 12.00ʺ E longitude) in Jammu and Kashmir, India. The sample collection was done as per the protocol standardized by Luster and co-workers^29^. The samples were collected in triplicate and stored at 4 °C.

### Media and growth conditions for *Fusarium oxysporum* strain R1

Previously isolated and characterised *Fusarium oxysporum* R1 (Genbank accession number KF663598) was used in this study^8^. The fungus was maintained on Potato Dextrose Agar at 25 °C for 72 h. The spore suspension (10^12^ spores ml^−1^) was prepared as per the protocol reported by Gupta and Vakhlu^8^.

### *Bacillus* species isolation

For isolation of *Bacillus* specifically from the cormosphere, microflora associated with corm sheath was screened. The soil adhered to corms was dusted by vigorous shaking and then the corm sheath (cormosphere) was separated from corm by peeling it off under sterile conditions. 1 gm of corm sheath was put in 10 ml normal saline and heated for 20 min at 80 °C to select *Bacillus.* This temperature is fatal to most of the non-spore forming bacteria. The 10^–6^ dilution of the suspension was spread on three media i.e. Luria–Bertani, Nutrient Agar and Minimal Media agar and incubated at 37 °C for 24 h. The isolated *Bacillus* species were purified by streak plate method and stored in 60% glycerol at − 80 °C till further screening^30^.

### Screening of isolated *Bacillus* strains for plant growth promotion (PGP) traits.

Isolated *Bacillus* species were evaluated in-vitro by qualitative as well as quantitative assays for various plant growth promoting and biocontrol properties. The PGP activities evaluated were indole acetic acid^31^, phosphate solubilization^32^, siderophore production^33^, amylase production^34^, protease production^35^ and antifungal activity against pathogenic *Fusarium oxysporum* R1^36^.

### Molecular characterization of *Bacillus* isolates

Genomic DNA of selected *Bacillus* strains was isolated using HiPurA bacterial genomic DNA purification kit (Himedia, India). The full length 16S ribosomal RNA gene was amplified using universal set primers, 8F (5′-AGAGTTTGATCCTGGCTCAG-3′) and 1522R (5′-AAGGAGGTGATCCANCCRCA-3′) primers^8^. The gene amplification was performed as per the modified protocol by Fierer and co-workers^37^ wherein, instead of 0.5 µM, 100 pM primer were used and PCR cycles were increased to 30 instead of 25 cycles. Initially DNA (50 ng) was denatured at 95 °C for 5 min followed by 30 cycles of denaturation at 95 °C for 1 min, annealing at 54 °C for 30 s and extension temperature was 72 °C for 1.5 min. The final extension was at 72 °C for 10 min. The amplicons were custom sequenced by SciGenom labs Pvt Ltd, Kerela, India. The nucleotide sequences obtained were assigned taxonomic affiliations, based on the closest match to the available sequences in the NCBI database (http://www.ncbi.nlm.nih.gov) using the EzTaxon version 2.1 (www.eztaxon.org). The molecular evolutionary and phylogenetic analysis was performed by aligning the sequences using multiple sequence alignment tools ClustalX 2.1 version followed by phylogenetic analysis by Phylip 3.69 (http://evolution.genetics.washington.edu/phylip.html) and MEGA X software^38^. The resulting sequences obtained were deposited in the GenBank nucleotide sequence database.

### Whole genome sequencing and annotation of *Bacillus* sp. strain D5

Genomic DNA of Bar D5 was isolated as mentioned above and sequenced using Ion torrent Personal Genome Machine (PGM) with 400 bp of read length at School of Biotechnology, University of Jammu, India. The raw reads were quality assessed using FastQC software^39^ and low quality reads (Q < 30) and adapters were removed. Good quality reads were de-novo assembled using ABySS version 3.10.1. Assembled genome was annotated using SEED and the Rapid Annotations using Subsystem Technology (RAST) version 2.0 server^40,41^. Coding sequences (CDS) were identified by RAST annotation. Genes responsible for plant growth promotion such as phosphate solubilization, siderophore production, iron acquisition, chemotaxis, chitin and N-acetyl glucosamine utilization were identified in the draft genome. For comparative genome analysis of Bar D5 strain, whole genome sequences of other *Bacillus* species*, Bacillus aryabhattai and Bacillus megaterium* strains were downloaded from NCBI. Burrows-Wheeler Aligner software^42^ and MAUVE software 2.4.0^43^, with progressive MAUVE algorithm were used for alignment of Bar D5 genome with reference genomes. *Bacillus megaterium* QMB1551 (NC_014019/CP001983), *Bacillus aryabhattai* B8W22 (NZ_FMZY0000000), *Bacillus aryabhattai* K13 (NZ_CP024035) and *Bacillus megaterium* DSM 319 (NC_014103/CP001982) were used as reference genomes for genome comparison.

### Genes for plant growth promotion

Genes responsible for plant growth promotion and biocontrol activity such as phosphorus solublization activity (mtnX), siderophore biosynthesis activity (asbA), ACC deaminase activity (acd), Indole pyruvate decarboxylase activity (ipdC), acid phosphatase production (yutF), induction of systemic resistance (yngG) and transcriptional regulation of bacillomycin D (degU)^44^ were selected based on the available literature to check their presence in Bar D5. The protein sequences were obtained from UniProt database and were reverse translated using tblastn database^45^. The resulting nucleotide sequences were then mapped on the draft genome of Bar D5 using CLC Sequence Viewer version 8.0. After mapping the genes present on the draft genome of Bar D5, specific primers were designed using Primer-BLAST tool^46^. The PCR program used for the amplification was denaturation for 5 min at 95 °C followed by 32 cycles of denaturation for 1 min at 95 °C, annealing temperature from 50 °C to 55 °C for 30 s and extension at 72 °C for 1.5 min and a final extension at 72 °C for 10 min. The resulting PCR products were sequenced at Sci genome Kerala, India.

### *Bacillus* sp. strain D5 based bio-formulation preparation

For inoculum preparation, 1 ml of 20 h old Bar D5 culture (log phase) was inoculated in 200 ml of nutrient broth in a 500 ml erlenmeyer flask and was incubated for 48 h at 35 ± 1 °C with shaking at 180 rpm. The Bar D5 reached stationary phase after 48 h under mentioned conditions and the cell count was 10^8^ CFU ml^−1^. 100 ml of Bar D5 broth (10^8^ CFU ml^−1^) was mixed with calcium carbonate in the ratio of 1:2 V/W respectively. The calcium carbonate (talc) was autoclaved twice at 121 °C for 15 min to make it sterile before mixing it with Bar D5 to form slurry. The slurry was dried for 4 days at 35–37 °C and finally 1% sterile CMC (Carboxymethyl cellulose) was added to it and stored at room temperature^26^. To study the viability of spores in bio-formulation, CFU was calculated for 1 year after every 30 days by serial dilution method to establish field/shelf life of bio-formulation^18^.

### In-vivo characterization of bio-formulation in pots

The effect of Bar D5 bio-formulation on growth parameters of saffron were evaluated in pot trials (with pot of 76 mm mouth diameter) as per complete randomized block design (CRBD). Sixty corms of uniform size (8 gm) were selected. A total of 30 corms were primed with Bar D5 bio-formulation (10^8^ CFU ml^−1^) and 30 corms were treated with only talc and CMC to serve as control^26^. The corms (control as well as treated) were planted in steam-sterilized sand: soil mixture (1:1 w/w) inoculated with *Fusarium oxysporum* R1 spores (10^12^ spores ml^−1^, 5 × 10^10^ spores gm^−1^ of soil) in pots, so as to evaluate disease incidence^28^. Pots were incubated in plant growth chamber, under 16 h of light and 8 h of dark cycle at 26 ± 2 °C with 80% relative humidity for 40 days, and were irrigated after every 3 days. Sixty pots were laid, thirty for control and thirty for treatment, maintaining one corm in each pot. The data collected was statistically analyzed.

### Field trials

#### Traditional area

The field trials were initiated in the month of September, 2016 in the Saffron growing fields of state agriculture department at Kishtwar, Jammu and Kashmir following complete randomized block design (CRBD). Rectangular raised beds of 4 feet × 6 feet with drainage channels and inter-bed distance of 3 feet on all the sides were prepared. For statistical relevance, around 648 corms were sown (324 corms as control and 324 corms Bar D5 primed). Three rectangular beds, each for control and treatment were made and approximately 108 corms (18 corms in each row) with 10 × 20 cm inter-corm and inter-row spacing were sown. The corms were sown at a depth of 13–15 cm from ground level^6^. Plant samples were collected during the flowering stage and vegetative stage of saffron life cycle.

#### Non traditional area

The field trials were laid in the month of August, 2018 at R.S Pura, (Jammu, Jammu and Kashmir), as per complete randomized block design (CRBD). Six rectangular beds, three for control and three for treated corms, of 3 feet × 4 feet in dimension were prepared and 50 corms (10 corms in each row) per bed were sown each for control and treatment with 10 × 20 cm inter-corm and inter row spacing. The corms were sown at a depth of 13–15 cm from ground level. Plant samples were collected during the flowering stage and vegetative stage of saffron life cycle.

### Statistical analysis

All the in-vivo experiments were conducted with three biological replicates and one biological replicate data consisted of mean of ten technical replicates. Results have been expressed as mean ± standard deviation (SD). Data was subjected to statistical analysis by analysis of variance (ANOVA) using IBM SPSS statistics version 26.

## Results

### Selection of potential *Bacillus* species with PGP and antifungal activity

**Figure 1:**
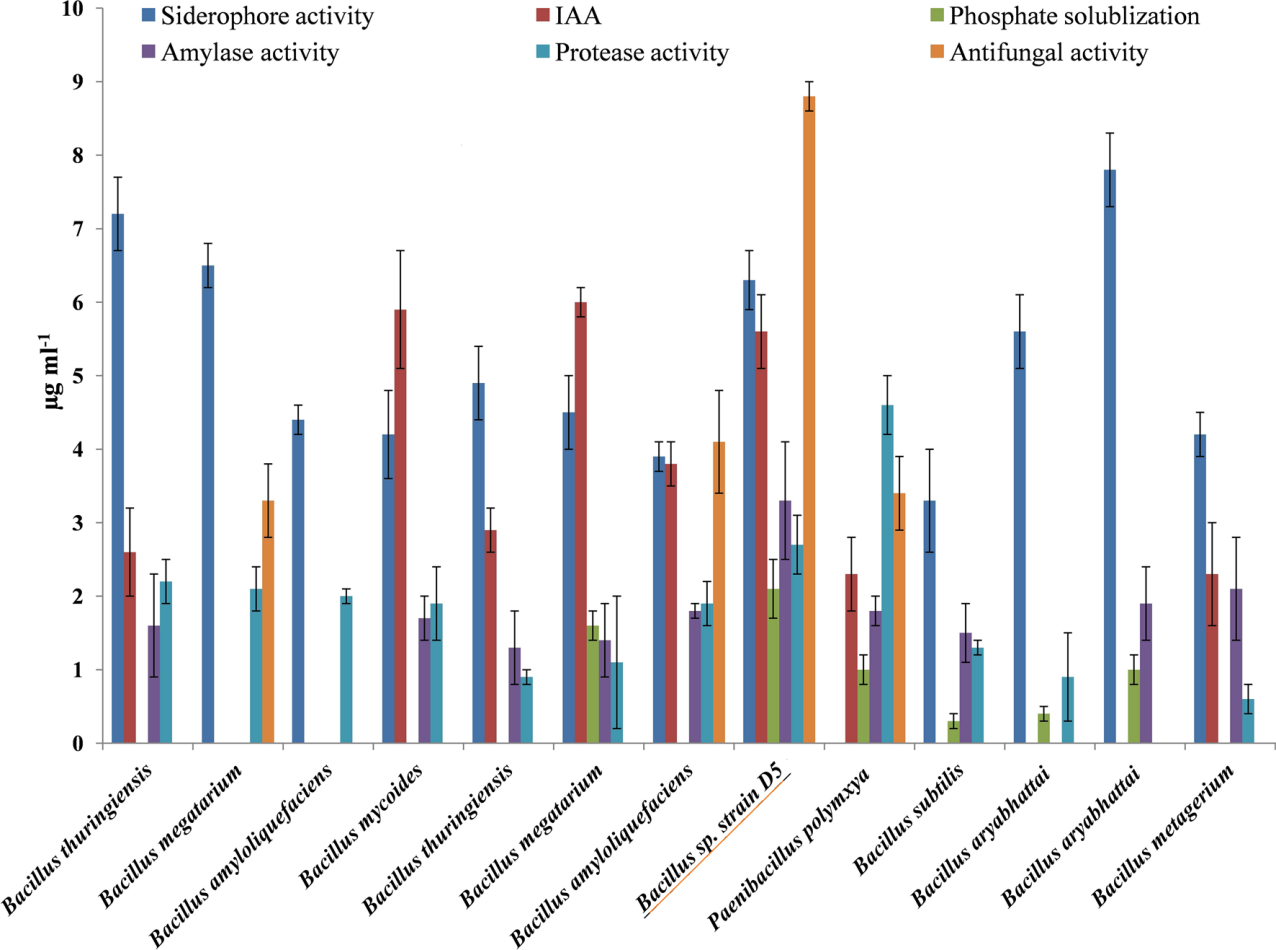
Functional profile of various *Bacillus* species isolate from cormposphere of saffron for various Plant growth promotion activities and antifungal activity against fungal pathogen *Fusarium oxysporum* R1 based upon this profile isolate D5 showed maximum potential to be used further in the study.

**Figure 2:**
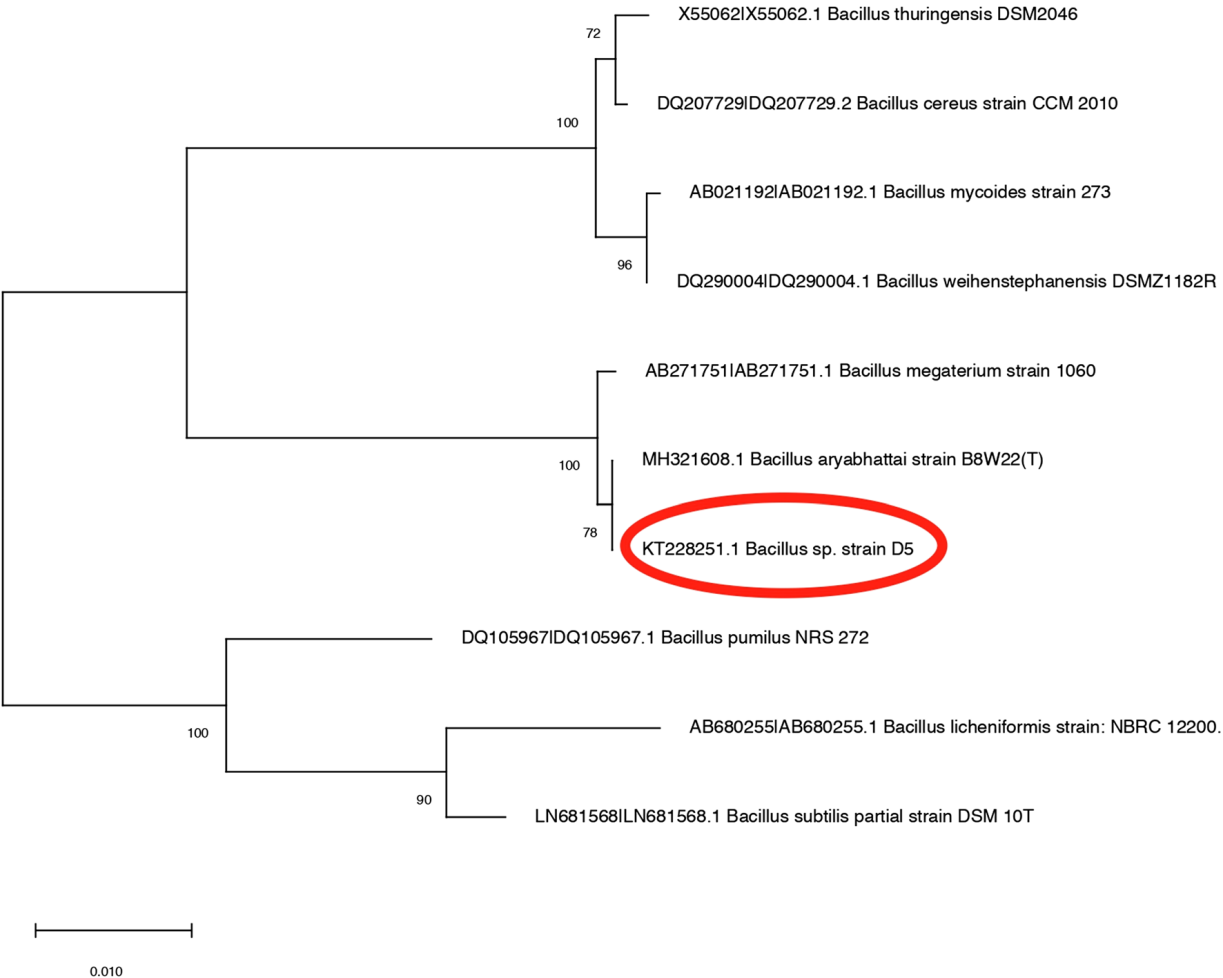
Evolutionary relationships of *Bacillus* sp. strain D5 with other known *Bacillus* type strains. The evolutionary tree was created using Neighbor-Joining method. The bootstrap concensus tree inferred from 1000 replicates. The evolutionary distances were computed using the Maximum Composite Likelihood method and are in the units of the number of base substitutions per site. Evolutionary analyses were conducted in MEGA X software.

The preliminary selection of *Bacillus* species was based on the initial heat treatment of corm sheath in normal saline solution at 80 °C. A total of 181 *Bacillus* morphotypes were isolated from cormosphere of the saffron from the three growth stages. All the 181 isolates were evaluated in-vitro for various PGP activities such as phosphate solubilization, indole acetic production, siderophore production, enzymes production (amylase and protease) and the inhibition of pathogenic *Fusarium oxysporum* R1 (Fox R1) on solid media. 13 shortlisted isolates had multiple PGP activity, out of which only four strains inhibited Fox R1 growth substantially i.e. with zone of inhibition ranging between of 3–9 mm Fig. 1, Table 1. All the 13 strains isolated were identified by 16S rRNA gene sequence similarity. The GenBank accession numbers of 13 strains has been listed in Table 1. *Bacillus* sp. with the best in-vitro PGP and antifungal activity against Fox R1 was identified as *Bacillus* sp. strain D5 (GenBank accession number KT228251) and was selected for further in-vivo evaluation in pots. The phylogenetic analysis of Bar D5 with other type strains of *Bacillus* species has been shown in Fig. 2.

**Table 1:** Quantitative analysis of plant growth promoting properties and antifungal potential of selected 13 short listed *Bacillus* species.

16S rRNA identification with strain	GenBank accession number	% similarity	Siderophore activity (μg/ml)	IAA (μg/ml)	Phosphate solublization (μg/ml)	Amylase activity (μg/ml)	Protease activity (μg/ml)	Antifungal activity (Zone of inhibition mm)
*Bacillus thuringiensis* DC1	KF702277	100	7.2 ± 0.5	2.6 ± 0.6	0	1.6 ± 0.7	2.2 ± 0.3	0
*Bacillus megaterium* DC2	KF702278	99	6.5 ± 0.3	0	0	0	2.1 ± 0.3	3.3 ± 0.5
*Bacillus amyloliquefaciens* DC8	KF702284	100	4.4 ± 0.2	0	0	0	2 ± 0.1	0
*Bacillus mycoides* DC7	KF702283	99	4.2 ± 0.6	5.9 ± 0.8	0	1.7 ± 0.3	1.9 ± 0.5	0
*Bacillus thuringiensis* FC6	KF702291	100	4.90.5	2.9 ± 0.3	0	1.3 ± 0.5	0.9 ± 0.1	0
*Bacillus megaterium* VC2	KF702293	99	4.5 ± 0.5	6 ± 0.2	1.6 ± 0.2	1.40.5	1.1 ± 0.9	0
*Bacillus amyloliquefaciens* VC5	KF702296	99	3.9 ± 0.2	3.8 ± 0.3	0	1.8 ± 0.1	1.9 ± 0.3	4.1 ± 0.7
*Bacillus* sp. strain D5	**KT228251**	**99**	**6.3 ± 0.4**	**5.6 ± 0.5**	**2.1 ± 0.4**	**3.3 ± 0.8**	**2.7 ± 0.4**	**8.8 ± 0.2**
*Paenibacillus polymxya* D7	KT228252	99	0	2.3 ± 0.5	1 ± 0.2	1.8 ± 0.2	4.6 ± 0.4	3.4 ± 0.5
*Bacillus subtilis* FR1O	KT228256	99	3.3 ± 0.7	0	0.30.1	1.5 ± 0.4	1.3 ± 0.1	0
*Bacillus aryabhattai* LB9	KT228263	99	5.6 ± 0.5	0	0.4 ± 0.1	0	0.9 ± 0.6	0
*Bacillus aryabhattai* LB17	KT228264	99	7.8 ± 0.5	0	1 ± 0.2	1.9 ± 0.5	0	0
*Bacillus metagerium* FR9	KT228255	99	4.2 ± 0.3	2.3 ± 0.7	0	2.1 ± 0.7	0.6 ± 0.2	0

### *Bacillus* sp. strain D5 based bio-formulation

**Figure 3:**
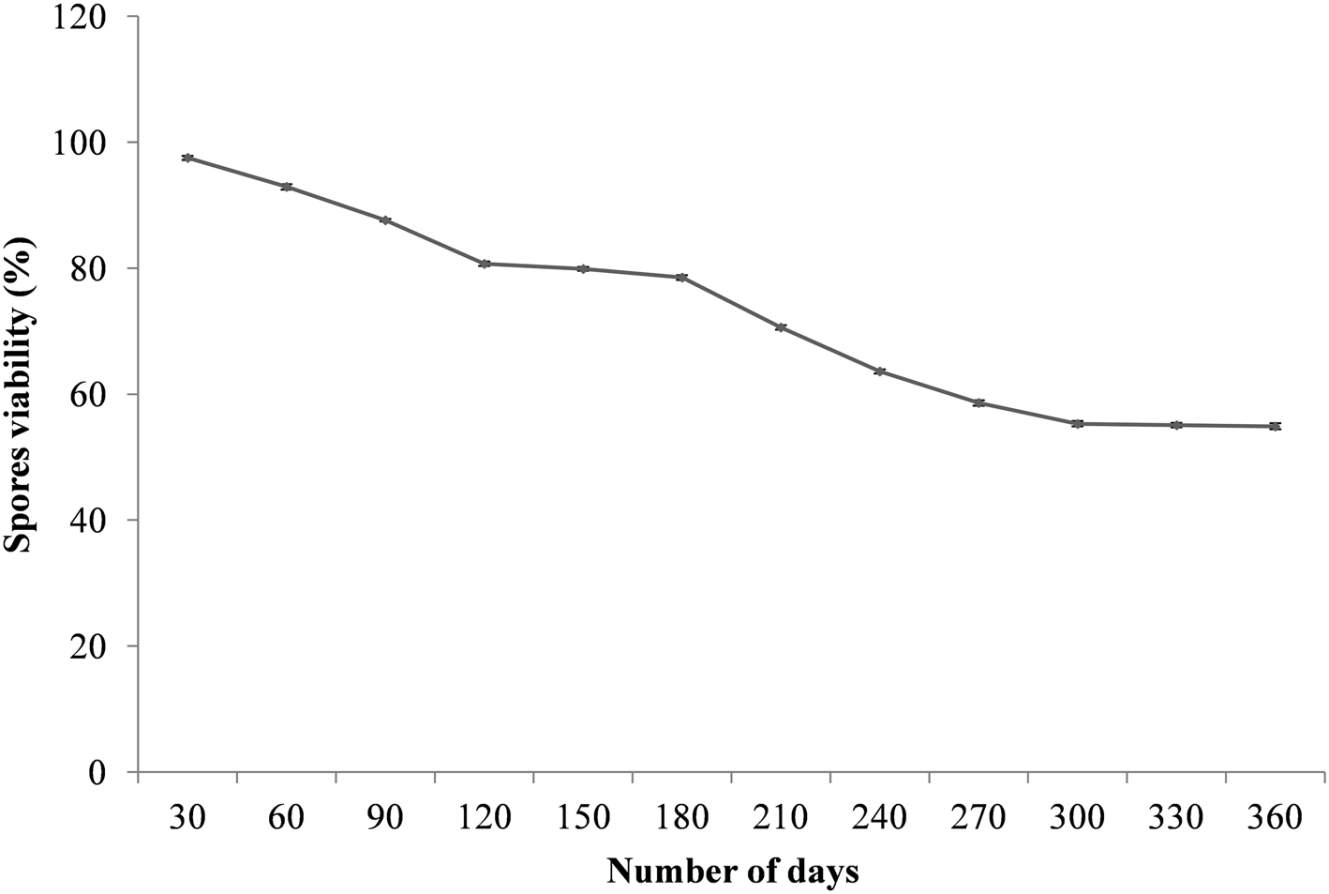
Viability of *Bacillus* sp. strain D5 spores when mixed with Calcium carbonate for the assessment of shelf-life at room temperature.

For *in-vivo* evaluation in the pots and fields, Bar D5 based bio-formulation was prepared using calcium carbonate in the ratio of 1:2. The bio-formulation was checked for viability of Bar D5 by calculating CFU up to 360 days. The 78% of Bar D5 spores were viable till 180 days and 52% were viable till one year at room temperature in the laboratory during various seasons Fig. 3.

### Evaluation of Bar D5 in pot assays

**Figure 4:**
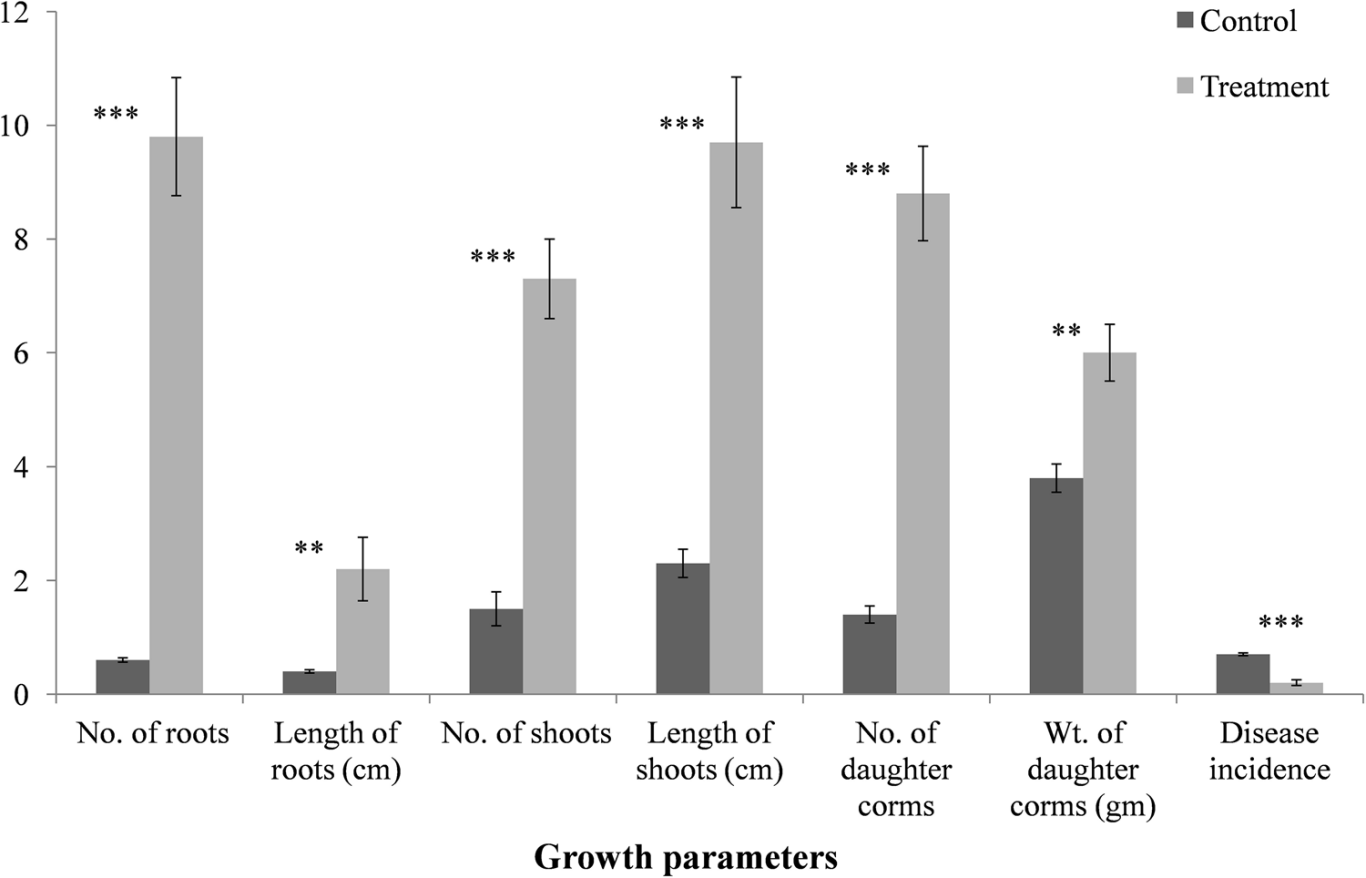
Effect of Bar D5 based bioformulation on saffron growth and disease control in Pot assay. Results are the average of ten replicates and experiments were performed in triplicates. Error bars represents the standard deviation. Corresponding results of ANOVA: *** represents the highly significant difference between means of control and treatment as P value (< 0.001), ** represents significant difference as P value (< 0.01).

Pot assays with Bar D5 treated corms resulted in significant increase in shoot–root length and shoot–root number. Number of roots increased to 9.8 ± 1.04 in treated corms as compared to 0.6 ± 0.04 in control, similarly number of shoots increased to 7.3 ± 0.7 in treated corms as compared to 1.5 ± 0.3 in control. The average length of roots was also higher in treated corms Treated (T) = 2.2 ± 0.56 cm as compared to control (C) = 0.4 ± 0.03 cm. Number and average weight of daughter corms in treated samples also increased significantly as compared to control (Number T = 8.8 ± 0.83, C = 1.4 ± 0.15; Average weight T = 6.0 ± 0.5 gm, C = 3.8 ± 0.25 gm). Importantly, the disease severity index was much less in treated corms as compared to control (T = 20%, C = 70%) Fig. 4, Table S1).

### Field evaluation of the* Bacillus* sp. strain D5 based bio-formulation

**Figure 5:**
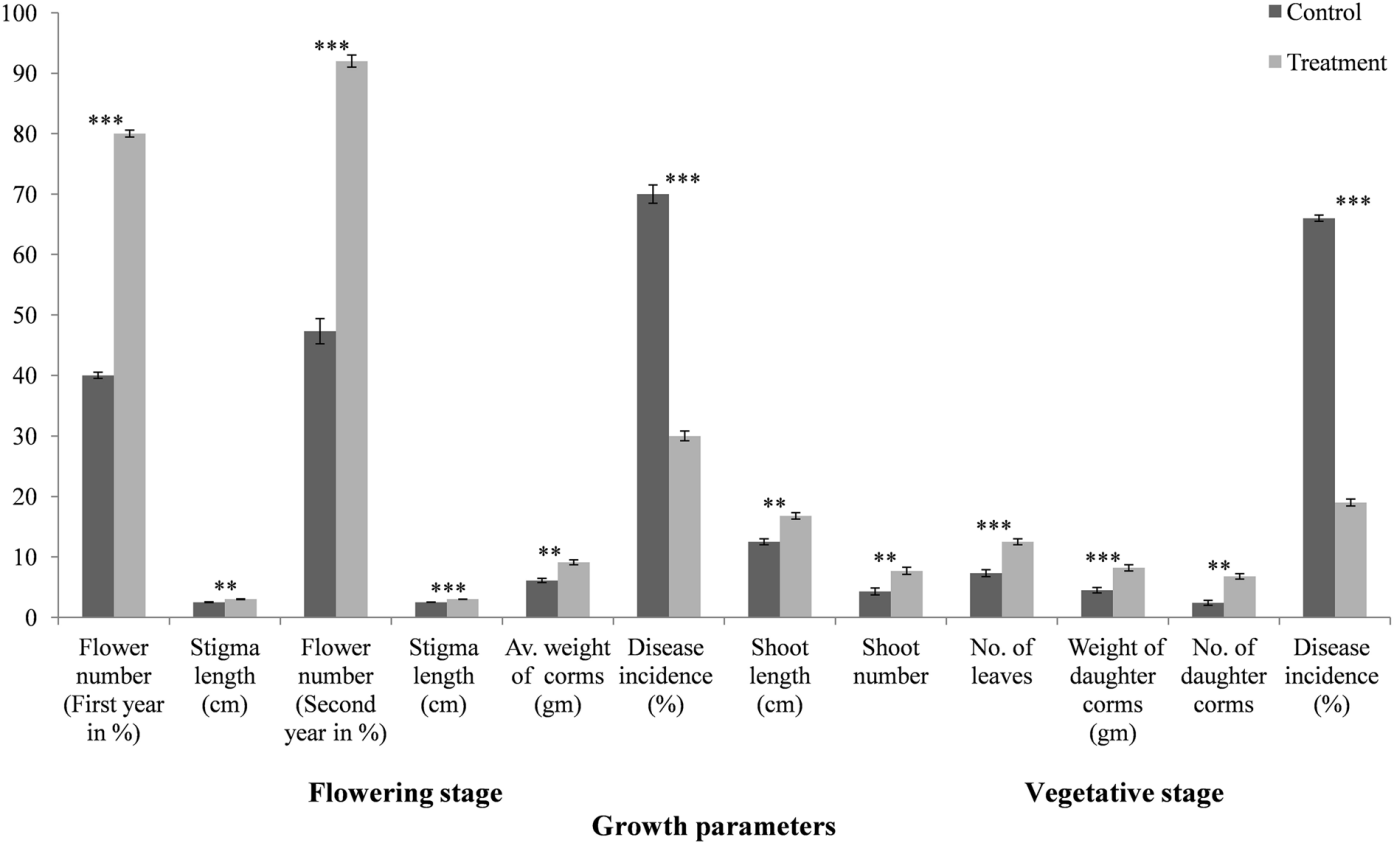
Evaluation of Bar D5 based bioformulation in the fields during the flowering and vegetatitive stage of saffron life cycle. Results are the average of ten replicates and experiments were performed in triplicates. Error bars represents the standard deviation. Corresponding results of ANOVA: *** represents the highly significant difference between means of control and treatment as P value (< 0.001), ** represents significant difference as P value (< 0.01).

The biocontrol and plant growth promoting effect of Bar D5 based bio-formulation was evaluated for two consecutive years in traditional saffron cultivation area i.e. Kishtwar fields, in Jammu, India (2016 to 2018). During the flowering stage, infection was 30% in treated corms as compared to 70% in control corms. Further, the number of flowers produced by treated samples was 246 from 308 corms sown (80%) as compared to the control 123 from 308 corms (40%). The length of stigma was 3 ± 0.05 cm in T and 2.5 ± 0.04 cm in C. The average weight of corms was T = 9.1 ± 0.4 gm and C = 6.1 ± 0.36 gm. Fig. 5, Table 2.

**Table 2:** Field evaluation of Bar D5 based bio-formulation during the flowering and vegetative stage of saffron life cycle.

	Growth parameters	Traditional area			ANOVA results		Non traditional area			ANOVA results	
		Control	Test	Fold change	F_1,4_ value	P value	Control	Test	Fold change	F_1,4_ value	P value
Flowering stage	Flower number (First year in %)	40 ± 0.50	80 ± 0.57	**2 ± 0.03**	7857.818	0.000	12 ± 0.57	22 ± 0.57	**1.76 ± 0.07**	392.000	0.000
	Stigma length (cm)	2.5 ± 0.05	3 ± 0.04	**1.2 ± 0.02**	81.869	0.001	2.2 ± 0.05	2.6 ± 0.07	**1.1 ± 0.03**	40.641	0.003
	Flower number (Second year in %)	47.3 ± 2.08	92 ± 1	**1.9 ± 0.10**	1122.250	0.000	24 ± 1.5	54 ± 1	**2.3 ± 0.20**	828.100	0.000
	Stigma length (cm)	2.5 ± 0.02	3 ± 0.01	**1.2 ± 0.02**	750.893	0.000	2.3 ± 0.03	2.7 ± 0.07	**1.2 ± 0.05**	76.970	0.001
	Weight of corm (gm)	6.1 ± 0.36	9.1 ± 0.4	**1.5 ± 0.03**	103.846	0.001	5.4 ± 0.05	8.6 ± 0.52	**1.6 ± 0.08**	103.953	0.001
	Disease incidence (%)	70 ± 1.5	30 ± 0.76	**2.3 ± 0.10↓**	1645.714	0.000	66 ± 1.5	35 ± 1	**1.9 ± 0.10↓**	883.600	0.000
Vegetative stage	Shoot length (cm)	12.5 ± 0.5	16.8 ± 0.52	**1.3 ± 0.02**	95.151	0.001	11.1 ± 0.76	14.2 ± 0.64	**1.2 ± 0.14**	28.926	0.006
	Shoot number	4.3 ± 0.57	7.7 ± 0.57	**1.8 ± 0.20**	50.000	0.002	3.4 ± 0.4	5.9 ± 0.32	**1.6 ± 0.07**	66.613	0.001
	Number of leaves	7.3 ± 0.57	12.5 ± 0.5	**1.7 ± 0.15**	137.286	0.000	7 ± 0.3	10.1 ± 0.36	**1.4 ± 0.16**	66.446	0.001
	Number of daughter corms	2.4 ± 0.40	6.8 ± 0.45	**2.8 ± 0.29**	91.562	0.001	2 ± 0.15	4.8 ± 0.35	**2.4 ± 0.30**	3192.350	0.000
	Weight of daughter corms (gm)	4.5 ± 0.45	8.23 ± 0.5	**1.8 ± 0.17**	153.223	0.000	2.9 ± 0.35	5 ± 0.25	**1.7 ± 0.29**	152.819	0.000
	Disease incidence	66 ± 0.50	19 ± 0.57	**3.4 ± 0.08↓**	10,961.286	0.000	59 ± 0.57	21 ± 1	**2.8 ± 0.16↓**	3192.250	0.000

Bold values highlight the infrence between the control and test sample.

**Figure 6:**
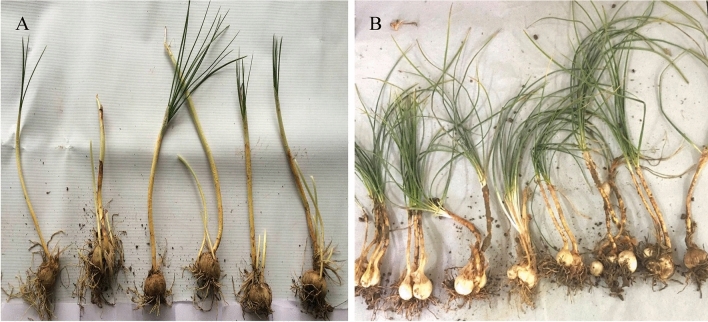
Comparison of plants samples obtained from the fields in Kishtwar in 2018. (**A**) Control samples and, (**B**) Plant samples treated with Bar D5 bio-formulation before sowing. The treated samples show better growth in terms of number and size of shoots and roots and production of daughter corms.

During the vegetative phase (when mother corm is consumed and gives rise to new daughter corms) results obtained suggest that there was considerable difference between the number of daughter corms produced by treated and control samples. The number and weight of daughter corms produced in treated samples were significantly enhanced as compared to control samples (Number, T = 6.8 ± 0.45 and C = 2.4 ± 0.4; Average weight, T = 8.2 ± 0.5 gm and C = 4.5 ± 0.45 gm). The disease incidence was also reduced to 19% in treated corms and to 66% in control samples. The shoot length and number were also higher in treated samples as compared to control samples (length, T = 16.8 ± 0.52 cm and C = 12.5 ± 0.50 cm; Number, T = 7.7 ± 0.57 and C = 4.3 ± 0.57). The number of leaves in treated sample was 12.5 ± 0.50 as compared to 7.3 ± 0.57 in control. The plant samples were uprooted from fields at the end of second year for comparative analysis and the treated plants were found to be healthier as compared to the control samples. The number of shoots and leaves was also higher in treated samples Fig. 6.

**Figure 7:**
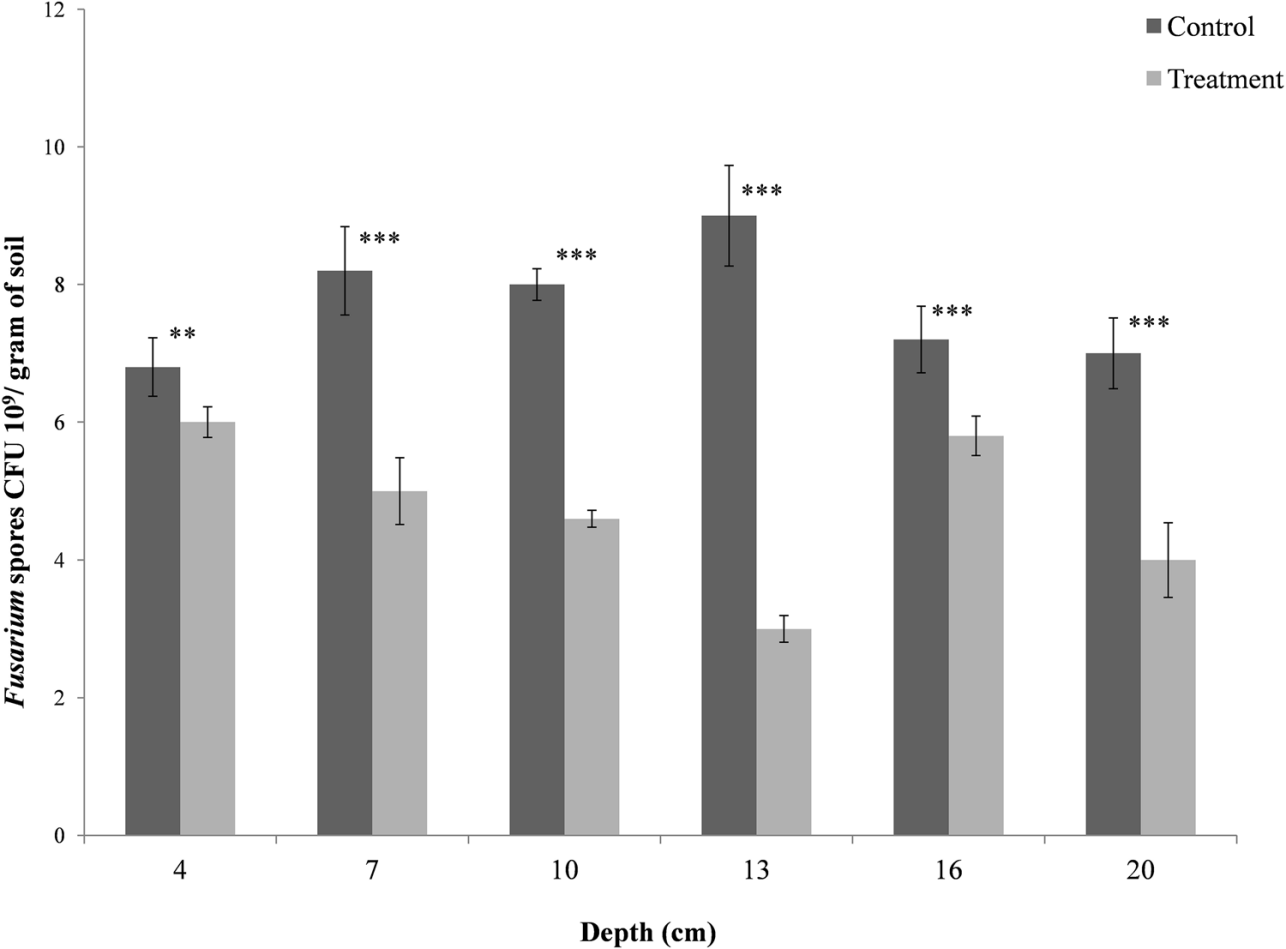
Analysis of fungal spores of at various depths in the control and treated field soil. Results are the average of five replicates. Error bars represents the standard deviation. Corresponding results of ANOVA: *** represents the highly significant difference between means of control and treatment as P value (< 0.001), ** represents significant difference as P value (< 0.01).

In order to study the effect of Bar D5 based bio-formulation on the fungal load, soil samples from various depths were compared for the presence of Fox R1 using *Fusarium* specific growth media. The study reveals that the soil sample at a depth of corm sowing (13–15 cm) had least number of *Fusarium* spores (3 × 10^9^ CFU gm^−1^ of soil) around Bar D5 primed corms as compared to the control soil (9 × 10^9^ CFU gm^−1^ of soil). Also, the *Fusarium* load was less at a depth of 13–15 cm as compared to soil at varying depth, indicating the potential role of Bar D5 in suppressing the growth of pathogenic fungus around the corms Fig. 7, Table S2).

**Figure 8:**
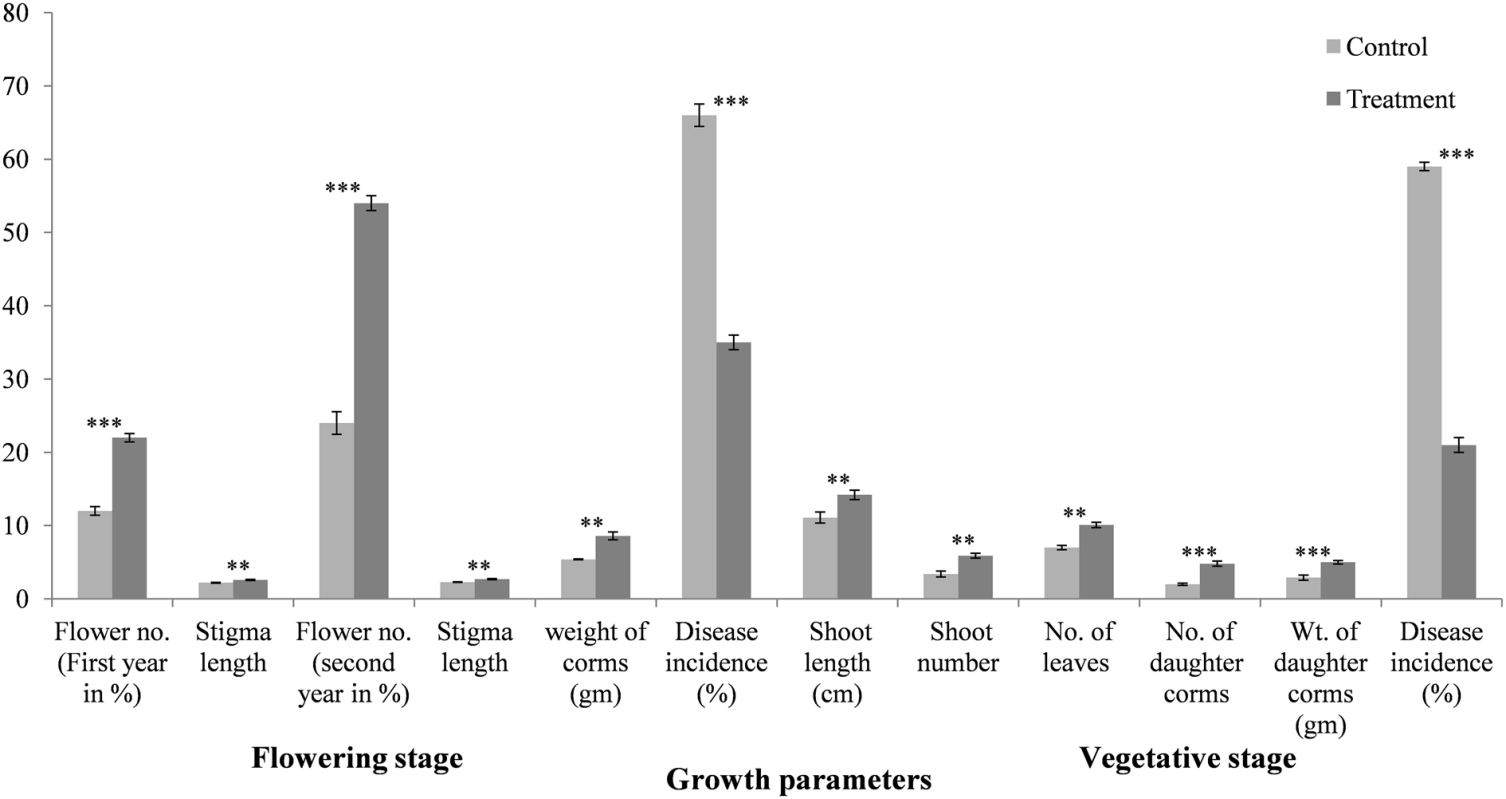
Evaluation of Bar D5 based bio**-**formulation on saffron growth during flowering and vegetatitive phase of life cycle in Jammu fields (Non traditional area of saffron cultivation). Results are the average of five replicates. Error bars represents the standard deviation. Corresponding results of ANOVA: *** represents the highly significant difference between means of control and treatment as P value (< 0.001), ** represents significant difference as P value (< 0.01).

Subsequently, in 2018–2019, plant growth promoting effect of Bar D5 based bio-formulation was also evaluated in fields of R.S. Pura Jammu, the non-traditional area of saffron cultivation. During the flowering stage in first year, out of 150 corms planted in each case, 33 flowers (22%) were produced in treated samples as compared to 18 flowers (12%) in control. The length of stigma was 2.6 ± 0.07 cm in treated and 2.2 ± 0.05 cm in control. Disease incidence was also less in treated samples (35%) as compared to control (66%). During vegetative stage, shoot length and number were also higher in treated plants (length T = 14.2 ± 0.64 cm and C = 11.1 ± 0.76 cm; Number T = 5.9 ± 0.3 and C = 3.4 ± 0.40). The number of leaves in treated samples was 10.1 ± 0.36 and in control was 7 ± 0.30. The total number and weight of daughter corms produced in treated samples was double as compared to the control samples (Number T = 0.4.8 ± 0.35 and C = 2 ± 0.15; Weight T = 5 ± 0.25 gm and C = 2.9 ± 0.35 gm). There was a significant difference in second year flowering percentage of saffron life cycle in non-traditional year. In treated samples, the flowering percentage was 54% (81 flowers from 150 corms planted) compared to control where it was only 24% (36 flowers from 150 corms planted) Fig. 8, Table 2.

A comparison of the outcome of field trials in traditional and non-traditional area has been tabulated in Table 2.

### Genome analysis of Bar D5

Whole genome sequencing of Bar D5 resulted in 250 Mb data with ~ 30 × coverage. Sequencing reads having more than Q30 phred score were used for de-novo assembly using ABySS version 3.10.1. De-novo assembly resulted in draft genome of ~ 3.9 Mb (3,959,600 bp) having 12 contigs with N_50_ as 3,726,54 bp and L_50_ as 4. The average G + C content of genome was 36.9%. Bar D5 draft genome annotation resulted in 4414 coding sequences (CDS), 65 RNAs and 349 subsystems as predicted by RAST annotation server. Various genes involved in cellular and metabolic processes of Bar D5 were annotated in the draft genome and their number has been tabulated in Table 3. All the genes responsible for plant growth promoting traits (that showed biochemical activity in plate based assay) were present in draft genome except genes for plant hormone indole acetic acid Table 3. Genes for PGP such as phosphorus metabolism (Table S3), iron acquisition (Table S4), siderophore metabolism (Table S5), for flagella biosynthesis and chemotaxis (Table S6) for chitin and N-acetyl glucosamine utilization (Table S7) were present in draft genome. In addition, genes for nitrogen metabolism, sulphur metabolism, potassium metabolism, genes for multiple drug resistance (resistance to fluoroquinnolones), heavy metal resistance (zinc, arsenic, cobalt-zinc-cadmium, cadmium), for beta lactamase enzymes, for stress response (osmotic stress, oxidative stress, cold shock, detoxification and carbon starvation), volatile compounds such as butanol biosynthesis and acetoin & butanediol metabolism, synthesis of bacteriocins and antibacterial peptides were also present Table 3. The whole-genome draft sequence has been deposited at DDBJ/ENA/GenBank under the accession number QGGC00000000. The version described here is QGGC01000000.

**Table 3:** Genes identified in the draft genome of Bar D5 involved in cellular and metabolic processes by RAST analysis.

S. no	Genes function	No. of genes involved
1	Cofactors, vitamins, prosthetic groups, pigments	120
2	Cell wall and capsule	120
3	Virulence, disease and defense	74
4	Potassium metabolism	16
5	Photosynthesis	0
6	Miscellaneous	27
7	Phages, prophages, transposable elements, plasmids	0
8	Membrane transport	54
9	Iron acquisition and metabolism	29
10	RNA metabolism	123
11	Nucleosides and nucleotides	60
12	Protein Metabolism	75
13	Cell division and cell cycle	3
14	Motility and chemotaxis	109
15	Regulation and cell signaling	44
16	Secondary metabolism	5
17	DNA metabolism	66
18	Fatty acids, lipids, and isoprenoids	64
19	Nitrogen metabolism	16
20	Dormancy and sporulation	40
21	Respiration	75
22	Stress response	81
23	Metabolism of aromatic compounds	16
24	Amino acids and derivatives	329
25	Sulfur metabolism	45
26	Phosphorus metabolism	42
27	Carbohydrates	334

**Figure 9:**
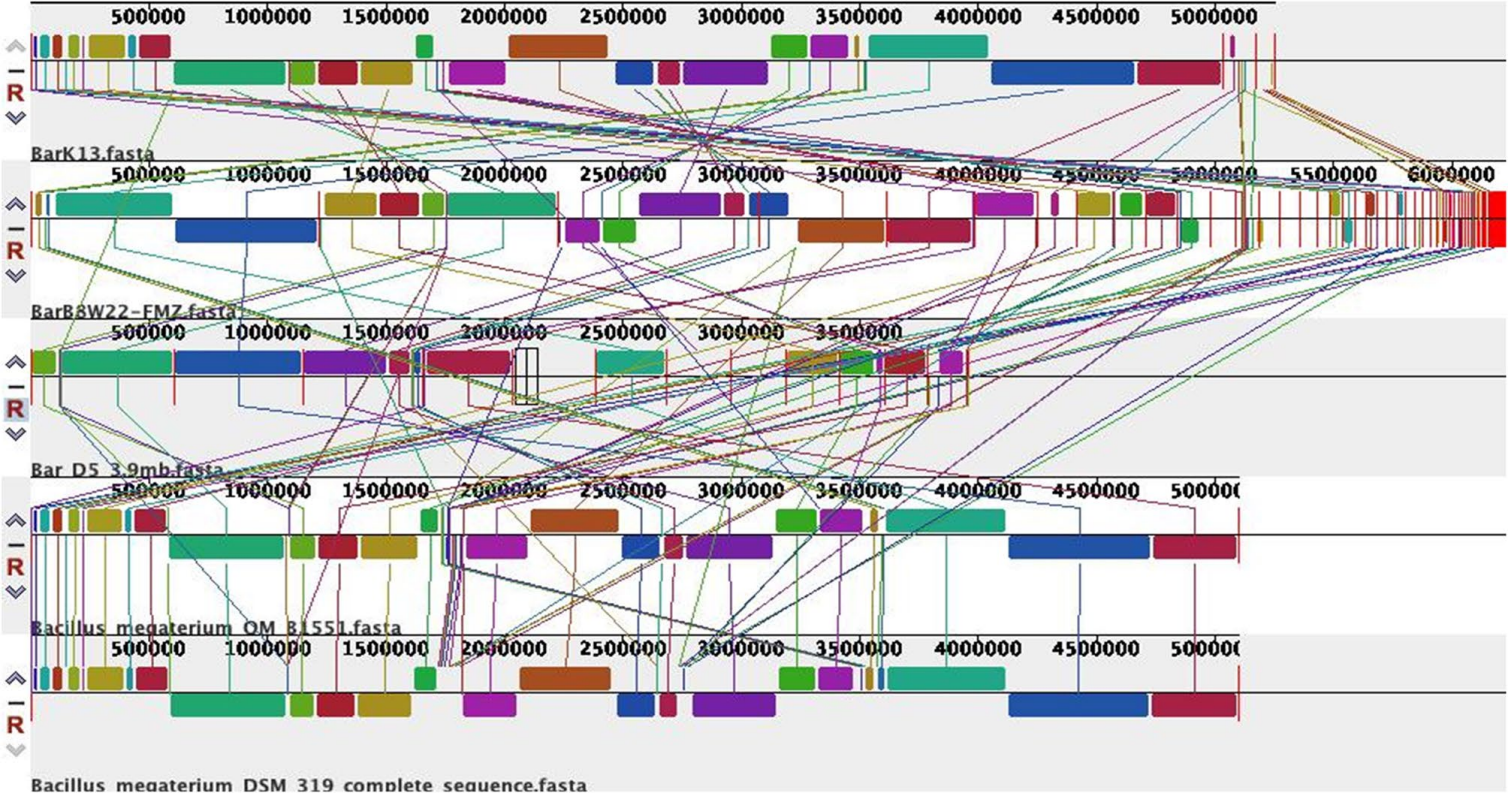
Comparison of genomes of Bar D5 in progressive MAUVE version 2.4.0 with *Bacillus aryabhattai* and *Bacillus megaterium* reference strains. Each genome panel contains the name of the genome sequence, a scale showing the sequence coordinates for that genome, and a single black horizontal centerline. Colored block outlines surround a region of the genome that aligned to part of another and is presumably homologous and internally free from genomic rearrangement.

RAST annotation predicted the top twenty closest neighboring strains of *Bacillus* sp. strain D5 wherein, *Bacillus megaterium* QMB1551 was the closest neighboring strain (Table S8). Though, 16S rRNA gene sequencing identified the *Bacillus* sp. as *Bacillus aryabhattai* and comparative genomic analysis using Burrows-Wheeler Aligner of *Bacillus* sp. strain D5 draft genome resulted in 99.47% similarity with both *Bacillus aryabhattai* B8W22 and *Bacillus aryabhattai* K13 and 100% with both *Bacillus megaterium* QMB1551 and *Bacillus megaterium* DSM319. Even, comparative genome analysis of Bar D5 with other 4 references (mentioned above) using MAUVE software also found that the draft genome of Bar D5 was equally similar to both *Bacillus aryabhattai* and *Bacillus megaterium.* This was well represented by conserved regions (colored regions in Fig. 9 in Bar D5 that were also present in all the other references Fig. 9. Coloured blocks are called Locally Collinear Blocks (LCB) that aligned among them in input genomes with same colored lines represent the regions of homology and internally free from genomic rearrangement.

### Confirmation of authenticity of draft genome by amplification and mapping of genes for PGP and biological control on draft genome of Bar D5

To confirm the authenticity of draft genome sequence, the PGP genetics determinants responsible for plant growth promotion activity and defense properties of *Bacillus* sp. strain D5 were PCR amplified, sequenced and re-mapped on the draft genome. In addition, the gene sequence for IAA was not identified by RAST analysis on draft genome but by amplification of the IAA from genome of Bar D5. The amplicon size obtained from these genes was 267 bp (mtnX), 381 bp (asbA), 369 bp (acd), 270 bp (ipdc), 367 bp (yutF), 255 bp (yngG) and 399 bp (degU). These amplicons were then sequenced and the resulting nucleotide sequences were mapped on the draft genome using CLC Sequence Viewer version 8.0 to reconfirm the position of genes on the draft genome and the GenBank accession number of these gene sequences has been tabulated in Table 4.

**Table 4:** Bar D5 genes for plant growth promotion and secondary metabolism amplified and mapped on draft genome.

S. no	Target gene	Primer Sequence	Gene function	Position on draft genome, (GenBank accession number)	Product length (bp)
1	**mtnX**	mtnX F1 AGATTGCATGAGCTTGCGGA mtnX R1 ACTGCACCCAGCATTCGTTA	Phosphorus solubilization	152,076–152,287 (QGGC01000011.1)	267
2	**asbA**	sbp F1 GCCAAGACCTTTTAGCAGCG sbp R1 ACGTCGGCTGAGTTACGTTT	Siderophore biosynthesis	137,935–138,248 (QGGC01000006.1)	381
3	**acd**	acdS F1 CATGCAGGATTAGTGGCAGGC acdS R1 TGAACCTCCTGAGTGGATAAACACC	ACC deaminase	4735–5061 (QGGC01000006.1)	369
4	**ipdC**	ipdC F2 GACGTCCCCACCATAATTAACAAA ipdC R2 CAGTGACCCAGAAGATGTAGCC	Indole pyruvate decarboxylase	82,725–82,494 (QGGC01000001.1)	270
5	**yutF**	yutF F1 CCGACATGCGTTGCTTGAAA yutF R1 GCCTGCATTCATCCCTGCTA	Acid phosphatase	322,241–322,552 (QGGC01000004.1)	367
6	**yngG**	yngG F1 AGGCGGTGGAAATGCAAGT yngG R1 AAGATGCTTCTACTGTTGCAAATAC	Induction of systemic resistance	485,264–485,456 (QGGC01000002.1)	255
7	**degU**	degU F1 TGCAGAAGGTGACGACGG degU R1 AACAAATGTAGTGGACGGCGA	Transcriptional regulation of Bacillomycin D	182,075–182,424 (QGGC01000004.1)	399

## Discussion

Saffron is the costliest spice in the world but with decline in its production and increased use in medicine & cosmetics, it has become the most adulterated spice as well^47,48^. The reasons for the yield loss are many but corm rot caused by various microbial pathogens and climate change are two of them^7,49^.

Out of 181 total *Bacillus* strains isolated from three growth phases of saffron, Bar D5 was comparatively better PGPB. Bar D5 based bio-formulation was also found to be better than previously reported three different bio-formulations by our group on the basis of pot assay evaluation Table 5. Earlier bio-formulations were (i) bacterial consortia comprising of (*Chryseobacterium elymi* WRF4, *Pseudomonas tremae* WRF2, *Acetinobacteria calcoaceticus* WRF1, *Bacillus aryabhattai* WRF5, *Pseudomonas kilonensis* WRF3 and *Pseudomonas koreensis* WRF6A^26^ (ii) *Bacillus amyloliquefaciens* W2^8^ and (iii) bacilli consortia comprising of *Bacillus thurinniensis* DC1, *Bacillus megaterium* VC3 and *Bacillus amyloliquefaciens* DC8^28^. Previously, reported *Bacillus aryabhattai* strains associated with saffron rhizosphere^26^ and cormosphere^28^ are from saffron fields in Kashmir. The one being reported in present study has been isolated from cormosphere of saffron plant grown in Kishtwar region of the state. The present strain Bar D5 is different from earlier reported *Bacillus aryabhattai* WRF5^26^ and *Bacillus aryabhattai* VC1^28^ as Bar WRF5 did not show phosphate solubilization activity and Bar VC1 did not show any antifungal activity.

**Table 5:** Comparative table for results of pot trials with different native bio-formulations.

Growth parameters	Column3mn4 Consortium 1 (*Acetinobacteria calcoaceticus* WRF1, *Pseudomonas tremae* WRF2, *Pseudomonas kilonensis* WRF3, *Chryseobacterium elymi* WRF4, *Bacillus aryabhattai* WRF5 and *Pseudomonas koreensis* WRF6A) (Ambardar and Vakhlu^5^)		Column6 Consortium 2 *(Bacillus thurinniensis* DC1, *Bacillus* megaterium VC3 and *Bacillus amyloliquefaciens* DC8) (Kour et al.^43^)		*Bacillus* sp. strain D5 alone (Present study)		*Bacillus amyloliquefaciens* W2 alone (Gupta and Vakhlu^30^)	
	Control	Test	Control	Test	Control	Test	Control	Test
Number of roots	1.2 ± 0.81	3.6 ± 1.82	0.6 ± 0.3	9.4 ± 2.8	0.6 ± 0.04	9.8 ± 1.04	–	–
Length of roots (cm)	0.1 ± 0.06	1.15 ± 0.54	0.6 ± 0.3	2.6 ± 0.1	0.4 ± 0.03	2.2 ± 0.56	–	–
Number of shoots	4.3 ± 0.53	5.6 ± 0.49	1 ± 0.5	4.2 ± 0.8	1.5 ± 0.3	7.3 ± 0.7	–	–
Length of shoots (cm)	9.95 ± 3.90	6.59 ± 1.59	1.4 ± 0.6	9 ± 1.9	2.3 ± 0.25	9.7 ± 1.15	–	–
Number of daughter corms	0.5 ± 0.4	3.9 ± 1.11	1.2 ± 0.2	6.4 ± 1.4	1.4 ± 0.15	8.8 ± 0.83	­	–
Disease incidence	0.6	0.4	0.6	0.2	0.7	0.2	2.8	1.2

*Bacillus* and *Pseudomonas* are used pre-dominantly as PGP and biological control agents, for both direct as well as indirect mechanism of plant growth promotion^50^. However, membres of *Bacillus* genera are preferred for bio-formulations preparation because of their long shelf/field life. Their long shelf life is due to their ability to form heat and desiccation-resistance spores, which can survive high temperatures and unsuitable pH^51,52^. Additionally, *Bacillus* also possesses several other pre-requisite PGP characteristics which includes their tendency to replicate at a faster rate, ability to colonise the roots rapidly and their competitive colonisation potential^53^. They promote the growth of plants by the production of various phytohormone precursors such as indole acetic acid, cytokinins, gibberellins and ethylene, conversion of complex nutrients like phosphorous and nitrogen in to simple forms, synthesis of siderophores, production of secondary metabolites and antifungal compounds^54,55^.

Bar D5 was characterized as plant growth promoting bacteria, as it produced siderophores, protease, indole acetic acid and solubilized phosphate that have important role in plant growth and disease control. Siderophores are low molecular weight compounds that chelate the iron compounds. Bacterial species producing these siderophores help in uptake of iron, which is a vital mineral nutrient required by the plants^56^. Siderophore producing bacterial isolates from cormosphere promote growth by making iron less available for the pathogenic species by iron chelation, hence act as biocontrol agents. Siderophore producing bacteria like *Bacillus* from the rhizosphere of *Piper nigrum* L. has been reported to have PGP activity on shallot bulbs and seeds of mustard^57^. Phosphate solubilizing bacteria helps in the uptake of phosphorous from soil and promotes plant growth^55^. Protease has been reported as a hydrolase which along with chitinase and β-1,3-glucanase causes fungal cell wall lysis, hence resulting in the disease control^8^. *Bacillus aryabhattai* has also been reported from *Erigeron Canadensis* and soybean with PGP activity^58,59^. *Bacillus aryabhattai* strain SRB02 promoted the growth of soyabean plant by modulating the production of various phytoharmones such as ABA, IAA, JA, GA12, GA4 and GA7^60^.

In addition to PGP activities, Bar D5 also has antifungal activity against pathogenic *Fusarium oxysporum* R1. *Bacillus* species, *B*. *subtilis* and *B*. *amyloliquefaciens* are well studied for their antagonistic activity towards *Fusarium* spp.^61^. *Bacillus amyloliquefaciens* FZB42 is a commercially available biocontrol agent and biofertilizer^62^. *Bacillus amyloliquefaciens* W2 has been reported as biological control agent against corm rot causing agent *Fusarium oxysporum* R1 in saffron^8^.

Bar D5 based bio-formulation has (52%) viable CFU up to 1 year at room temperature Fig. 3 and was evaluated in pots and fields by corms priming. Jyoti and co-workers^63^ have studied the viability of consortia (*Providencia vermicola* A2 and *Klebsiella pneumoniae* CP19) in talc based and bagasse based bio-formulation and was found to be stable for 70 days. Viability of endophytic *Bacillus* sp. CaB5 was investigated in talc based bio-formulation by plate count method & fluorescence method and was found to be stable up to 45 days^64^. The decline in viability with time can be due to nutrient depletion and autolysis of cells^18^.

Pot trails with bio-formulation resulted in significant increase in the various parameters contributing to plant growth promotion wherein root number, shoot number, number of daughter cormlets was enhanced by 17.7 folds, 4.9 folds and 6.6 folds respectively whereas corm rot disease was decreased by 3.6 folds as compared to control. Park and co-workers^60^ have reported *B*. *aryabhattai* SRB02 from soyabean rhizosphere where it significantly increased the root and shoot length of plant in pots by the production of various phytoharmones. Similarly, *Bacillus aryabhattai* strain AB211, reported from maize, also significantly increased the root/shoot length, dry and wet weight of maize seedlings in pot experiments by solubilizing inorganic phosphorus, synthesized siderophore and producing IAA^65^. Brahim et al.^66^ reported *Bacillus* sp. BCLRB2 with multiple PGP properties (IAA, chitinase & protease production and phosphate solubilization) that increased the maize length by 31% and fresh biomass by 43% under saline conditions in pot assays. *Bacillus subtilis* EA-CB0575 from banana rhizosphere increased the total dry weight (root + shoot) of banana plant by 34.60% under green house conditions^67^. In the present study, the increase in root-shoot length and number attributed to the production of phytoharmone IAA, phosphate solubilization and siderophore production by Bar D5 making inslouble nutrients present in soil, available to plant and increase in cormlets production could be due to starch hydrolysis and IAA production by *Bacillus* strain^28^. The starch hydrolysis in saffron corm helps the floral, vegetative buds and roots to differentiate during dormancy. The starch in saffron corms is converted into sucrose and other suitable sugars and transported to the tissues, where buds are differentiated and developed^68^. Similarly in maize, IAA producing *Bacillus* strains have been reported to improve seed germination and seedling growth^57^.

The field evaluation of bio-formulation further increased flower number by 2 folds, stigma length by 1.2 folds and reduced disease incidence by 2.3 folds in treated as compared to control corms. Along with disease suppression, treated plants produced more daughter corms (2.83 folds) and were healthy than the control sample during vegetative phase Fig. 6. The reduction of disease in the fields due to the competition for the niche, siderophore production, and induction of plant immune system that activates hyper-defense response upon pathogen attack (Ali et al. Un published data). Bar D5 treated corms showed significant increased in all the growth parameters in flowering and vegetative stage in non-traditional area as well. In flowering stage, bio-formulation increased the flower number by 1.7 folds, stigma length by 1.1 folds and reduced the disease incidence by 1.9 folds. The weight of corms was 1.6 folds more in treated corms compared to control. In vegetative stage also, there was significant difference in growth parameters such as shoot length (1.2 folds), shoot number (1.6 folds), number of daughter corms (2.4 folds) as well as reduction in disease incidence (2.8 folds). Similar to present study, *Bacillus Xiamenensis* PM14 isolated from sugarcane rhizosphere exhibited various PGP activities (phosphate solubiltion, siderophore production, IAA production catalase, protease, chitinase, pectinase, cellulose) increased the fresh weight of plant by 30%, root length by 37%, plant length by 5%, cane length by 27.5% and significantly reduced the incidence of red rot disease in green house experiments^69^.

Xiang and co-workers^70^ found that *Bacillus subtilis* subsp. *Subtilis* strains Bsssu2, BSSSu3 and *B. velezensis* strain Bve12 increased the soya bean plant height and biomass in the green house trials and *Bacillus altitudinis* stain Bal13 increased early growth of soya bean in green house and microplot trials. Also, in field trials, *Bacillus safensis* strain Bsa27 and (*B. velezensis* starin Bve2 + *Bacillus altitudinis* strain Bal13) reduced *Heterodera glycines* cyst population density at 60 DAP. PGPR *Bacillus velezensis* Ba168 has been reported as promising biological control agent against tobacco black shank disease caused by pathogen *Phytophthora nicotianae,* which reduced the disease index in field assays^71^. Recently, Karthika et al.^72^ isolated bacterium *Bacillus cereus* KTMA4 from tomato rhizosphere which was found to be effective against phytopathogens *Fusarium oxysporum* and *Alternaria saloni* and also, increased the plant height, fresh/wet weight in green house experiments.

In present study, the effect of the bio-formulation on the length of stigma, in addition to the increase in corm and flower number is commercially very important. Recently, Zhou et al.^73^ has drawn co-relation between root-shoot development and stigma quality in saffron. The hypothesis can be proposed that increase in shoot–root length and number induced by Bar D5 could be the reason for longer stigma length and higher number of corms and flowers. In depth studies are needed to be conducted to understand the underlying mechansim.

In order to mine the genes responsible for plant growth promotion and biocontrol potential, whole genome of *Bacillus* sp. strain D5 was sequenced using Ion torrent PGM technology and the genome was assembled de-novo, by using ABySS version 3.10. The draft genome of Bar D5 (3,959,600 bp) was found to be smaller than the closely related *Bacillus megaterium* QMB1551 (5,097,129 bp) and *Bacillus megaterium* DSM319 (5,097,447 bp) genome^74^. Draft genome sequence of Bar D5 revealed the presence of PGP genes in the genome, reported to be responsible for biocontrol activity and growth promotion. Presence of genes responsible for motility and chemotaxis (Table S6) indicated that Bar D5 is plant associated bacterium, as these genes are responsible for bacterial movement towards the plant roots in response to the exudates and other nutrients released by the plants^75^. Presence of genes for iron acquisition (Table S4), siderophore synthesis (Table S5) and antibiotic resistance usually help the strain by protecting it from antibiotics produced by other organisms and by inhibiting pathogen by iron chelation^65^. Presence of genes for phosphate solubilization, ammonia assimilation, sulphur metabolism, potassium metabolism revealed that the Bar D5 has plant growth promotion potential. The presence of genes for stress response (cold stress, carbon starvation) demonstrates that it can survive harsh environmental conditions as well. Bar D5 genome has all genes for the production of volatile compounds (acetoin & 2,3-butanediol). Volatile compounds produced by root associated bacteria are known to elicit induced systemic resistance in plants^76^*.* Ryu and co-workers^77^, for the first time reported the role of *Bacillus* volatile compounds in plant growth and induction of systemic resistance. *Bacillus subtilis* GB03 and *B. amyloliquefaciens* IN937a capable of promoting plant growth and induction of systemic resistance utilizing volatile compounds such as 3-hydroxy-2-butanone (acetoin) and 2,3-butanediol^77^. *Bacillus subtilus* SYST2, capable of producing volatile compounds (albuterol and 1,3-propanediole) with plant growth promotion activity has already been reported^78^. Volatile compounds produced by *Bacillus* sp. also inhibits the growth of various fungal pathogens, hence act as antifungal compounds^79^.

Phylogenetic analysis by 16S rRNA gene indicated that *Bacillus* sp. strain D5 has maximum similarity with *Bacillus aryabhattai* B8W22 type strain but RAST annotated draft genome showed maximum similarity with *Bacillus megaterium* QMB1551. In order to confirm the taxonomy at species level, genome alignment of Bar D5 was performed against four reference genome were done using Burrows-Wheeler Aligner software, which showed 100% similarity with *Bacillus megaterium* QMB1551 and *Bacillus megaterium* DSM319 whereas 99.47% similarity with *Bacillus aryabhattai* B8W22 and *Bacillus aryabhattai* K13. Comparative genome analysis using MAUVE software also depicted that the Bar D5 was equally similar to both *Bacillus megaterium* and *Bacillus aryabhattai* represented by the conserved regions common in all the species. Though , *Bacillus aryabhattai* and *Bacillus megaterium* are closely related species but no conclusion was drawn regarding species of the *Bacillus* under study as genome of Bar D5 is a draft genome with gaps in it Therefore, at present it was identified taxonomically only at genus level and referred as *Bacillus* sp. strain D5. Ray and co workers^80^ and Bhattacharyya and co workers^65^ have also reported the close homology of *Bacillus megaterium* with *Bacillus aryabhattai* suggesting common evolutionary relationship of the two species of *Bacillus*. Gap filling and closing the genome sequence requires re-sequencing, preferably on multiple platforms, which will be the future course of present study.

Most of the PGP genes were present in the draft genome except for genes responsible for plant growth hormone, IAA though it showed IAA activity biochemically on plate based assay. To reconfirm the presence of gene for IAA production and other PGP activities, fresh amplification of PGP genes were done from the genome of Bar D5. The selection of genes for reconfirmation was done based on the already reported bacterial plant growth promotion and antifungal genes^44^. The selected genes were mapped on the draft genome of Bar D5 and specific primers were designed for the amplification of Bar D5 genes Table 4. Since, all the basic genes necessary for plant growth promotion and biocontrol activity were present in Bar D5 genome, it further confirms its potential for commercial scale. An additional feature of being native to saffron plant makes it more worthy as it will not affect natural microflora associated with saffron corms. Therefore there are less chances of development of resistant pathotypes.

## Conclusion

The gap in production and demand of saffron, in international market is huge. However, its yield is decreasing due to corm rot disease and climate change. Use of chemical fertilizers and antifungal agents to increase production is not sustainable for obvious reasons. Saffron is a sustainable crop, as it requires low inputs and water but is labour intensive in terms of harvesting and post harvesting processing. Traditionally, it is cultivated in Iran, Spain, India, Italy, France, Switzerland, Morocco and India but now its cultivation is extended to USA, Australia, China and Afghanistan etc. The reason is profitability on account of its application in medicine, food and cosmetics. Therefore, there is international market for alternative to chemical agents, for augmentation of saffron production, and this is going to increase. Bar D5 based bio-formulation is being proposed as one such alternative. The field data in present study has been complimented with the genomic data of the *Bacillus*, with PGP genes sequenced and mapped on draft genome, indicating that the PGP properties of Bar D5 are genetic and hence stable.

## Supplementary Information

Supplementary Information
