# Supplementary material for: Field evaluation of PGP Bacillus sp. strain D5 native to Crocus sativus, in traditional and non traditional areas, and mining of PGP genes from its genome
Source: Sci Rep. 2021 Mar 9;11:5454. doi: 10.1038/s41598-021-84585-z (PMC7943801; doi:10.1038/s41598-021-84585-z)
Supplement: Supplementary file 1 — Supplementary Information [file 41598_2021_84585_MOESM1_ESM.pdf]

Field evaluation of PGP *Bacillus* sp. strain D5 native to *Crocus sativus*, in traditional and non traditional areas, and mining of PGP genes from its genome

Shanu Magotra[a,e#], Nancy Bhagat[a#], Sheetal Ambardar[b], Tahir Ali[a], Barbara Reinhold Hurek[c], Thomas Hurek[c], Praveen Kumar Verma[d]* and Jyoti Vakhlu[a]*

[a]Metagenomic Laboratory, School of Biotechnology, University of Jammu, India

[b]National Center for Biological Sciences, Bellary Road, Bangalore 560065, India

[c]Department of Microbe-Plant Interactions, Faculty of Biology and Chemistry, University of Bremen, Germany

[d]Plant Immunity Laboratory, National Institute of Plant Genome Research (NIPGR), New Delhi, India

[e]University Institute of Biotechnology, Chandigarh University, Punjab, India

# both authors have equal contribution

[c]*Corresponding author:

Dr. Praveen Kumar Verma

Scientist VI

National Institute of Plant Genome Research (NIPGR), New Delhi, 110067

Email: pkv@nipgr.ac.in

[a]*Corresponding author:

Prof. JyotiVakhlu

Metagenomic Laboratory,

School of Biotechnology,

University of Jammu

Email:jyotimetagenomic@gmail.com

Table S1: Effect of Bar D5 based bioformulation on saffron growth and disease control in Pot assays.

Growth parameters	Control	Treatment	$F_{1,4}$ value	p value
**Number of roots**	0.6±0.04	9.8±1.04	235.296	0.000
**Length of roots (cm)**	0.4±0.03	2.2±0.56	32.649	0.005
**Number of shoots**	1.5±0.3	7.3±0.7	156.250	0.000
**Length of shoots (cm)**	2.3±0.25	9.7±1.15	118.471	0.000
**No. of daughter corms**	1.4±0.15	8.8±0.83	248.191	0.000
**Weight of daughter corms (gm)**	3.8±0.25	6.0±0.5	47.755	0.002
**Disease incidence**	0.7	0.2	525.625	0.000

Based on Anova results, all the values are statistically significant as p value < 0.01.

Table S2: Analysis of fungal spores at various depths in the control and treated field soil.

Depth (cm)	Control	Treated samples	$F_{1,8}$ value	P value
**4**	6.8±0.43	6±0.22	13.913	0.006
**7**	8.2±0.65	5±0.48	91.733	0.000
**10**	8±0.23	4.6±0.12	827.530	0.000
**13**	9±0.73	3±0.19	396.826	0.000
**16**	7.2±0.49	5.8±0.29	31.804	0.000
**20**	7±0.51	4±0.54	73.130	0.000

Based on Anova results, all the values are statistically significant as p value < 0.01.

Table S3: Phosphorus metabolism genes mined on the draft genome of Bar D5 by RAST analysis.

S.No.	Contig ID/ Length	Start codon	Stop codon	Length (bp)	Function	Subsystem	Sub category
1	NODE_10_len_223748_cov_32.035_ID_19	129901	128642	1260	Alkaline phosphatase (EC 3.1.3.1)	Phosphate metabolism	Phosphorus metabolism- no category
2	NODE_11_len_197250_cov_35.3146_ID_21	169475	168066	1410	Phosphate regulon sensor protein PhoR (SphS) (EC 2.7.13.3)	High affinity phosphate transporter and control of PHO regulon, PhoR-PhoB two-component regulatory system, Phosphate metabolism	Phosphorus metabolism- no category
3	NODE_13_len_171125_cov_27.8357_ID_25	32027	31323	705	Phosphate regulon transcriptional regulatory protein PhoB (SphR)	High affinity phosphate transporter and control of PHO regulon, PhoR-PhoB two-component regulatory system, Phosphate metabolism	Phosphorus metabolism- no category
4	NODE_2_len_608628_cov_33.798_ID_3	602331	601714	618	Alkaline phosphatase like protein	Phosphate metabolism	Phosphorus metabolism- no category
5	NODE_3_len_539618_cov_36.6284_ID_5	137599	138336	738	Alkaline phosphatase synthesis transcriptional regulatory protein PhoP	High affinity phosphate transporter and control of PHO	Phosphorus metabolism- no category

						regulon,<br>Phosphate metabolism	
6	NODE_3_len_539618_cov_36.6284_ID_5	138329	140101	1773	Phosphate regulon sensor protein PhoR (SphS) (EC 2.7.13.3)	High affinity phosphate transporter and control of PHO regulon,<br>PhoR-PhoB two-component regulatory system,<br>Phosphate metabolism	Phosphorus metabolism-no category
7	NODE_3_len_539618_cov_36.6284_ID_5	196595	197593	999	Probable low-affinity inorganic phosphate transporter	Phosphate metabolism	Phosphorus metabolism-no category
8	NODE_3_len_539618_cov_36.6284_ID_5	286092	286979	888	Phosphate transport system permease protein PstC (TC 3.A.1.7.1)	High affinity phosphate transporter and control of PHO regulon,<br>Phosphate metabolism	Phosphorus metabolism-no category
9	NODE_3_len_539618_cov_36.6284_ID_5	286976	287905	930	Phosphate transport system permease protein PstA (TC 3.A.1.7.1)	High affinity phosphate transporter and control of PHO regulon,<br>Phosphate metabolism	Phosphorus metabolism-no category
10	NODE_3_len_539618_cov_36.6284_ID_5	349257	350231	975	Phosphate starvation-inducible protein PhoH, predicted ATPase	CBSS-56780.10.peg.1536,<br>CBSS-56780.10.peg.1536,<br>Phosphate metabolism,<br>Phosphate metabolism	Phosphorus metabolism-no category
11	NODE_3_len_539618_cov_36.6284_ID_5	383592	384401	810	Phosphate transport ATP-binding protein PstB (TC	High affinity phosphate transporter	Phosphorus metabolism-

					3.A.1.7.1)	and control of PHO regulon,<br>Phosphate metabolism	no category
12	NODE_3_len_539618_cov_36.6284_ID_5	384424	385083	660	Phosphate transport system regulatory protein PhoU	High affinity phosphate transporter and control of PHO regulon,<br>Phosphate metabolism	Phosphorus metabolism-no category
13	NODE_4_len_509767_cov_34.4692_ID_7	237142	237786	645	Alkaline phosphatase synthesis transcriptional regulatory protein PhoP	High affinity phosphate transporter and control of PHO regulon,<br>Phosphate metabolism	Phosphorus metabolism-no category
14	NODE_4_len_509767_cov_34.4692_ID_7	237808	239283	1476	Phosphate regulon sensor protein PhoR (SphS) (EC 2.7.13.3)	High affinity phosphate transporter and control of PHO regulon,<br>PhoR-PhoB two-component regulatory system,<br>Phosphate metabolism	Phosphorus metabolism-no category
15	NODE_5_len_372654_cov_35.0448_ID_9	43199	41301	1899	Alkaline phosphatase (EC 3.1.3.1)	Phosphate m	Phosphorus metabolism-no category
16	NODE_8_len_273711_cov_22.2621_ID_15	91370	92779	1410	Predicted ATPase related to phosphate starvation-inducible protein PhoH	Phosphate metabolism	Phosphorus metabolism-no category
17	NODE_2_len_608628_cov_33.798_ID_3	436079	434238	1842	2-aminoethylphosphonate:pyruvate aminotransferase (EC 2.6.1.37)	Phosphoenolpyruvate phosphomutase	Phosphorus metabolism-no category

18	NODE_2_len_608628_cov_33.798_ID_3	436721	436164	558	Phosphonopyruvate decarboxylase (EC 4.1.1.82)	Phosphoenolpyruvate phosphomutase	Phosphorus metabolism- no category
19	NODE_2_len_608628_cov_33.798_ID_3	437206	436718	489	Phosphonopyruvate decarboxylase (EC 4.1.1.82)	Phosphoenolpyruvate phosphomutase	Phosphorus metabolism- no category
20	NODE_2_len_608628_cov_33.798_ID_3	437363	437199	165	Phosphoenolpyruvate phosphomutase (EC 5.4.2.9)	Phosphoenolpyruvate phosphomutase	Phosphorus metabolism- no category
21	NODE_2_len_608628_cov_33.798_ID_3	437826	437314	513	Phosphoenolpyruvate phosphomutase (EC 5.4.2.9)	Phosphoenolpyruvate phosphomutase	Phosphorus metabolism- no category
22	NODE_2_len_608628_cov_33.798_ID_3	438718	437912	807	Phosphoenolpyruvate phosphomutase (EC 5.4.2.9)	Phosphoenolpyruvate phosphomutase	Phosphorus metabolism- no category

Table S4: List of genes for Iron acquisition in Bar D5

S.No.	Contig ID/ Length	Start codon	Stop codon	Length(bp)	Function	Subsystem	Sub category
1	NODE_11_len_197250_cov_35.3146_ID_21	36137	35442	696	Heme efflux system permease HrtA	Heme, hemin uptake and utilization systems in GramPositives	Iron acquisition and metabolism- no sub category
2	NODE_11_len_197250_cov_35.3146_ID_21	37222	36137	1086	Heme efflux system permease HrtB	Heme, hemin uptake and utilization systems in GramPositives	Iron acquisition and metabolism- no sub category
3	NODE_11_len_197250_cov_35.3146_ID_21	38677	37295	1383	Sensor histidine kinase colocalized with HrtAB transporter	Heme, hemin uptake and utilization systems in GramPositives	Iron acquisition and metabolism- no sub category
4	NODE_11_len_197250_cov_35.3146_ID_21	39348	38674	675	Two-component response regulator colocalized with HrtAB transporter	Heme, hemin uptake and utilization systems in GramPositives	Iron acquisition and metabolism- no sub category
5	NODE_2_len_608628_cov_33.798_ID_3	337941	336925	1017	Heme ABC type transporter HtsABC, permease protein HtsC	Heme, hemin uptake and utilization systems in GramPositives	Iron acquisition and metabolism- no sub category

6	NODE_2_len_608628_cov_33.798_ID_3	339909	339061	849	Heme ABC type transporter HtsABC, heme-binding protein	Heme, hemin uptake and utilization systems in GramPositives	Iron acquisition and metabolism- no sub category
7	NODE_2_len_608628_cov_33.798_ID_3	340006	339872	135	Heme ABC type transporter HtsABC, heme-binding protein	Heme, hemin uptake and utilization systems in GramPositives	Iron acquisition and metabolism- no sub category
8	NODE_4_len_509767_cov_34.4692_ID_7	196273	197556	1284	Heme ABC transporter, ATPase component HmuV	Heme, hemin uptake and utilization systems in GramPositives	Iron acquisition and metabolism- no sub category
9	NODE_5_len_372654_cov_35.0448_ID_9	32055	32768	714	Two-component response regulator SA14-24	Heme, hemin uptake and utilization systems in GramPositives, SA:14-24	Iron acquisition and metabolism- no sub category
10	NODE_5_len_372654_cov_35.0448_ID_9	32775	34682	1908	Two-component sensor kinase SA14-24	Heme, hemin uptake and utilization systems in GramPositives, SA:14-24	Iron acquisition and metabolism- no sub category
11	NODE_5_len_372654_cov_35.0448_ID_9	281715	281002	714	Ferrous iron transport permease EfeU	Ferrous iron transporter EfeUOB, low-pH-induced	Iron acquisition and metabolism- no sub category

Table S5: List of genes for siderophore synthesis in Bar D5 genome

S.No.	Contig ID/ Length	Start codon	Stop codon	Length(bp)	Function	Subsystem	Sub category
1	NODE_10_len_223748_cov_32.035_ID_19	119176	120186	1011	ABC-type Fe3+-siderophore transport system, permease component	Siderophore assembly kit	Siderophore
2	NODE_2_len_608628_cov_33.798_ID_3	269365	268271	1095	Siderophore biosynthesis diaminobutyrate--2-oxoglutarate aminotransferase (EC 2.6.1.76)	DAP (1,3-diaminopropane) production, Siderophore assembly kit	Siderophore
3	NODE_2_len_608628_cov_33.798_ID_3	269589	269419	171	Siderophore biosynthesis diaminobutyrate--2-oxoglutarate aminotransferase (EC 2.6.1.76)	DAP (1,3-diaminopropane) production, Siderophore assembly kit	Siderophore
4	NODE_2_len_608628_cov_33.798_ID_3	338981	337947	1035	ABC-type Fe3+-siderophore transport system, permease component	Siderophore assembly kit	Siderophore
5	NODE_5_len_372654_cov_35.0448_ID_9	292968	292141	828	ABC-type Fe3+-siderophore transport system, periplasmic iron-binding component	Siderophore assembly kit	Siderophore
6	NODE_5_len_372654_cov_35.0448_ID_9	293303	294313	1011	ABC-type Fe3+-siderophore transport system, permease component	Siderophore assembly kit	Siderophore
7	NODE_5_len_372654_cov_35.0448_ID_9	294310	295359	1050	ABC-type Fe3+-siderophore transport	Flavohaemoglobin, Siderophore	Siderophore

					system, permease 2 component	assembly kit	
8	NODE_5_len_372654_cov_35.0448_ID_9	295386	296213	828	ABC-type Fe3+-siderophore transport system, ATPase component	Siderophore assembly kit	Siderophore
9	NODE_7_len_295108_cov_36.1261_ID_13	138988	137183	1806	Siderophore synthetase superfamily, group C @ Siderophore synthetase component, ligase	Siderophore assembly kit	Siderophore
10	NODE_7_len_295108_cov_36.1261_ID_13	140142	138985	1158	Siderophore related permease	Siderophore assembly kit	Siderophore
11	NODE_7_len_295108_cov_36.1261_ID_13	141435	140146	1290	Siderophore biosynthesis protein, monooxygenase	Siderophore assembly kit	Siderophore
12	NODE_7_len_295108_cov_36.1261_ID_13	142007	141414	594	Siderophore synthetase small component, acetyltransferase	Siderophore assembly kit	Siderophore
13	NODE_7_len_295108_cov_36.1261_ID_13	143758	142004	1755	Siderophore synthetase superfamily, group A @ Siderophore synthetase large component, acetyltransferase	Siderophore assembly kit	Siderophore
14	NODE_7_len_295108_cov_36.1261_ID_13	146623	145259	1365	Siderophore biosynthesis diaminobutyrate--2-oxoglutarate aminotransferase (EC 2.6.1.76)	DAP (1,3-diaminopropane) production, Siderophore assembly kit	Siderophore

Table S6: Genes for motility and chemotaxis in Bar D5 genome

S.No.	Contig ID/ Length	Start codon	Stop codon	Length (bp)	Function	Subsystem	Sub category
1	NODE_10_len_223748_cov_32.035_ID_19	95389	94535	855	Chemotaxis protein methyltransferase CheR (EC 2.1.1.80)	Bacterial Chemotaxis	Motility and chemotaxis- no subcategory
2	NODE_6_len_357440_cov_16.0934_ID_11	107410	107655	246	Chemotaxis protein CheC -- inhibitor of MCP methylation	Bacterial Chemotaxis	Motility and chemotaxis
3	NODE_6_len_357440_cov_16.0934_ID_11	107723	108016	294	Chemotaxis protein CheC -- inhibitor of MCP methylation	Bacterial Chemotaxis	Motility and chemotaxis
4	NODE_6_len_357440_cov_16.0934_ID_11	108016	108501	486	Chemotaxis protein CheD	Bacterial Chemotaxis	Motility and chemotaxis
5	NODE_6_len_357440_cov_16.0934_ID_11	325865	325488	378	Flagellar motor switch protein FliN	Bacterial Chemotaxis, Flagellar motility, Flagellum	Motility and chemotaxis
6	NODE_6_len_357440_cov_16.0934_ID_11	326674	326108	567	Flagellar motor switch protein FliN	Bacterial Chemotaxis, Flagellar motility, Flagellum	Motility and chemotaxis
7	NODE_7_len_295108_cov_36.1261_ID_13	258307	257813	495	Chemotaxis protein CheD	Bacterial Chemotaxis	Motility and chemotaxis
8	NODE_7_len_295108_cov_36.1261_ID_13	258785	258357	429	Positive regulator of CheA protein activity (CheW)	Bacterial Chemotaxis	Motility and chemotaxis
9	NODE_7_len_295108_cov_36.1261_ID_13	269756	269394	363	Chemotaxis regulator - transmits	Bacterial Chemotaxis,	Motility and chemotaxis

					chemoreceptor signals to flagelllar motor components CheY	Flagellar motility	
10	NODE_7_len_295108_cov_36.1261_ID_13	270908	269778	1131	Flagellar motor switch protein FliN	Bacterial Chemotaxis, Flagellar motility, Flagellum	Motility and chemotaxis
11	NODE_7_len_295108_cov_36.1261_ID_13	271899	270913	987	Flagellar motor switch protein FliM	Bacterial Chemotaxis, Flagellar motility, Flagellum	Motility and chemotaxis
12	NODE_8_len_273711_cov_22.2621_ID_15	17489	18406	918	Chemotaxis protein CheV (EC 2.7.3.-)	Bacterial Chemotaxis, Flagellar motility	Motility and chemotaxis
13	NODE_8_len_273711_cov_22.2621_ID_15	30235	29870	366	Chemotaxis regulator - transmits chemoreceptor signals to flagelllar motor components CheY	Bacterial Chemotaxis, Flagellar motility	Motility and chemotaxis
14	NODE_8_len_273711_cov_22.2621_ID_15	80444	81259	816	Chemotaxis response regulator protein-glutamate methylesterase CheB (EC 3.1.1.61)	Bacterial Chemotaxis	Motility and chemotaxis
15	NODE_13_len_171125_cov_27.8357_ID_25	170067	168736	1332	Cell division protein FtsI [Peptidoglycan synthetase] (EC 2.4.1.129)	CBSS-83331.1.peg.3039, Flagellum in Campylobacter, Peptidoglycan Biosynthesis	Flagellar motility in Prokaryota

16	NODE_2_len_608628_cov_33.798_ID_3	135853	136764	912	Flagellin protein FlaA	Flagellum, Flagellum in Campylobacter	Flagellar motility in Prokaryota
17	NODE_7_len_295108_cov_36.1261_ID_13	282074	281784	291	Flagellar hook-basal body complex protein FliE	Flagellum, Flagellum in Campylobacter	Flagellar motility in Prokaryota
18	NODE_7_len_295108_cov_36.1261_ID_13	282536	282087	450	Flagellar basal-body rod protein FlgC	Flagellum, Flagellum in Campylobacter	Flagellar motility in Prokaryota
19	NODE_7_len_295108_cov_36.1261_ID_13	282706	282539	168	Flagellar basal-body rod protein FlgB	Flagellum, Flagellum in Campylobacter	Flagellar motility in Prokaryota
20	NODE_8_len_273711_cov_22.2621_ID_15	32462	31644	819	Flagellin protein FlaA	Flagellum, Flagellum in Campylobacter	Flagellar motility in Prokaryota
21	NODE_8_len_273711_cov_22.2621_ID_15	33527	32721	807	Flagellin protein FlaA	Flagellum, Flagellum in Campylobacter	Flagellar motility in Prokaryota
22	NODE_8_len_273711_cov_22.2621_ID_15	53574	54380	807	Flagellin protein FlaA	Flagellum, Flagellum in Campylobacter	Flagellar motility in Prokaryota
23	NODE_8_len_273711_cov_22.2621_ID_15	82498	83322	825	Flagellin protein FlaA	Flagellum, Flagellum in Campylobacter	Flagellar motility in Prokaryota
24	NODE_13_len_171125_cov_27.8357_ID_25	170067	168736	1332	Cell division protein FtsI [Peptidoglycan synthetase] (EC 2.4.1.129)	CBSS-83331.1.peg.3039, Flagellum in Campylobacter, Peptidoglycan Biosynthesis	Flagellar motility in Prokaryota
25	NODE_2_len_608628_cov_33.798_ID_3	135853	136764	912	Flagellin protein FlaA	Flagellum, Flagellum in	Flagellar motility in

						Campylobacter	Prokaryota
26	NODE_7_len_295108_cov_36.1261_ID_13	282074	281784	291	Flagellar hook-basal body complex protein FliE	Flagellum, Flagellum in Campylobacter	Flagellar motility in Prokaryota
27	NODE_7_len_295108_cov_36.1261_ID_13	282536	282087	450	Flagellar basal-body rod protein FlgC	Flagellum, Flagellum in Campylobacter	Flagellar motility in Prokaryota
28	NODE_3_len_539618_cov_36.6284_ID_5	85033	85257	225	Flagellar motor rotation protein MotA	Flagellar motility, Flagellum	Flagellar motility in Prokaryota
29	NODE_3_len_539618_cov_36.6284_ID_5	85223	85825	603	Flagellar motor rotation protein MotA	Flagellar motility, Flagellum	Flagellar motility in Prokaryota
30	NODE_3_len_539618_cov_36.6284_ID_5	85860	86474	615	Flagellar motor rotation protein MotB	Flagellar motility, Flagellum	Flagellar motility in Prokaryota
31	NODE_5_len_372654_cov_35.0448_ID_9	249187	250497	1311	RNA polymerase sigma-54 factor RpoN	Flagellar motility, Flagellum, Transcription initiation, bacterial sigma factors	Flagellar motility in Prokaryota
32	NODE_6_len_357440_cov_16.0934_ID_11	325865	325488	378	Flagellar motor switch protein FliN	Bacterial Chemotaxis, Flagellar motility, Flagellum	Flagellar motility in Prokaryota
33	NODE_6_len_357440_cov_16.0934_ID_11	326674	326108	567	Flagellar motor switch protein FliN	Bacterial Chemotaxis, Flagellar motility, Flagellum	Flagellar motility in Prokaryota
34	NODE_7_len_295108_cov_36.1261_ID_13	257757	256996	762	RNA polymerase sigma factor for flagellar operon	Flagellar motility, Flagellum, Transcription	Flagellar motility in Prokaryota

						initiation, bacterial sigma factors	
35	NODE_7_len_295108_cov_36.1261_ID_13	262733	261864	870	Flagellar synthesis regulator FleN	Flagellar motility, Flagellum	Flagellar motility in Prokaryota
36	NODE_7_len_295108_cov_36.1261_ID_13	263823	262726	1098	Flagellar biosynthesis protein FlhF	Flagellar motility, Flagellum	Flagellar motility in Prokaryota
37	NODE_7_len_295108_cov_36.1261_ID_13	265859	263823	2037	Flagellar biosynthesis protein FlhA	Flagellar motility, Flagellum	Flagellar motility in Prokaryota
38	NODE_7_len_295108_cov_36.1261_ID_13	266942	265890	1053	Flagellar biosynthesis protein FlhB	Flagellar motility, Flagellum	Flagellar motility in Prokaryota
39	NODE_7_len_295108_cov_36.1261_ID_13	267743	266976	768	Flagellar biosynthesis protein FliR	Flagellar motility, Flagellum	Flagellar motility in Prokaryota
40	NODE_7_len_295108_cov_36.1261_ID_13	269756	269394	363	Chemotaxis regulator - transmits chemoreceptor signals to flagelllar motor components CheY	Bacterial Chemotaxis, Flagellar motility	Flagellar motility in Prokaryota
41	NODE_7_len_295108_cov_36.1261_ID_13	270908	269778	1131	Flagellar motor switch protein FliN	Bacterial Chemotaxis, Flagellar motility, Flagellum	Flagellar motility in Prokaryota
42	NODE_7_len_295108_cov_36.1261_ID_13	271899	270913	987	Flagellar motor switch protein FliM	Bacterial Chemotaxis, Flagellar motility, Flagellum	Flagellar motility in Prokaryota
43	NODE_3_len_539618_cov_36.6284_ID_5	85033	85257	225	Flagellar motor	Flagellar motility,	Flagellar

					rotation protein MotA	Flagellum	motility in Prokaryota
44	NODE_3_len_539618_cov_36.6284_ID_5	85223	85825	603	Flagellar motor rotation protein MotA	Flagellar motility, Flagellum	Flagellar motility in Prokaryota
45	NODE_3_len_539618_cov_36.6284_ID_5	85860	86474	615	Flagellar motor rotation protein MotB	Flagellar motility, Flagellum	Flagellar motility in Prokaryota
46	NODE_5_len_372654_cov_35.0448_ID_9	249187	250497	1311	RNA polymerase sigma-54 factor RpoN	Flagellar motility, Flagellum, Transcription initiation, bacterial sigma factors	Flagellar motility in Prokaryota
47	NODE_6_len_357440_cov_16.0934_ID_11	325865	325488	378	Flagellar motor switch protein FliN	Bacterial Chemotaxis, Flagellar motility, Flagellum	Flagellar motility in Prokaryota
48	NODE_6_len_357440_cov_16.0934_ID_11	326674	326108	567	Flagellar motor switch protein FliN	Bacterial Chemotaxis, Flagellar motility, Flagellum	Flagellar motility in Prokaryota
49	NODE_7_len_295108_cov_36.1261_ID_13	257757	256996	762	RNA polymerase sigma factor for flagellar operon	Flagellar motility, Flagellum, Transcription initiation, bacterial sigma factors	Flagellar motility in Prokaryota
50	NODE_7_len_295108_cov_36.1261_ID_13	262733	261864	870	Flagellar synthesis regulator FleN	Flagellar motility, Flagellum	Flagellar motility in Prokaryota
51	NODE_7_len_295108_cov_36.1261_ID_13	263823	262726	1098	Flagellar biosynthesis protein FlhF	Flagellar motility, Flagellum	Flagellar motility in Prokaryota

52	NODE_7_len_295108_cov_36.1261_ID_13	265859	263823	2037	Flagellar biosynthesis protein FlhA	Flagellar motility, Flagellum	Flagellar motility in Prokaryota
53	NODE_7_len_295108_cov_36.1261_ID_13	266942	265890	1053	Flagellar biosynthesis protein FlhB	Flagellar motility, Flagellum	Flagellar motility in Prokaryota
54	NODE_7_len_295108_cov_36.1261_ID_13	267743	266976	768	Flagellar biosynthesis protein FliR	Flagellar motility, Flagellum	Flagellar motility in Prokaryota
55	NODE_7_len_295108_cov_36.1261_ID_13	271899	270913	987	Flagellar motor switch protein FliM	Bacterial Chemotaxis, Flagellar motility, Flagellum	Flagellar motility in Prokaryota
56	NODE_7_len_295108_cov_36.1261_ID_13	273848	273420	429	Flagellar basal-body rod modification protein FlgD	Flagellar motility, Flagellum	Flagellar motility in Prokaryota
57	NODE_7_len_295108_cov_36.1261_ID_13	278355	277042	1314	Flagellum-specific ATP synthase FliI	Flagellar motility, Flagellum	Flagellar motility in Prokaryota
58	NODE_8_len_273711_cov_22.2621_ID_15	12519	13310	792	Flagellar motor rotation protein MotA	Flagellar motility, Flagellum	Flagellar motility in Prokaryota
59	NODE_8_len_273711_cov_22.2621_ID_15	17489	18406	918	Chemotaxis protein CheV (EC 2.7.3.-)	Bacterial Chemotaxis, Flagellar motility	Flagellar motility in Prokaryota
60	NODE_8_len_273711_cov_22.2621_ID_15	30235	29870	366	Chemotaxis regulator - transmits chemoreceptor signals to flagelllar motor components CheY	Bacterial Chemotaxis, Flagellar motility	Flagellar motility in Prokaryota
61	NODE_13_len_171125_cov_27.8357_ID_25	170067	168736	1332	Cell division protein	CBSS-	Flagellar

					FtsI [Peptidoglycan synthetase] (EC 2.4.1.129)	83331.1.peg.3039, Flagellum in Campylobacter, Peptidoglycan Biosynthesis	motility in Prokaryota
62	NODE_2_len_608628_cov_33.798_ID_3	135853	136764	912	Flagellin protein FlaA	Flagellum, Flagellum in Campylobacter	Flagellar motility in Prokaryota
63	NODE_7_len_295108_cov_36.1261_ID_13	282074	281784	291	Flagellar hook-basal body complex protein FliE	Flagellum, Flagellum in Campylobacter	Flagellar motility in Prokaryota
64	NODE_7_len_295108_cov_36.1261_ID_13	282536	282087	450	Flagellar basal-body rod protein FlgC	Flagellum, Flagellum in Campylobacter	Flagellar motility in Prokaryota
65	NODE_7_len_295108_cov_36.1261_ID_13	282706	282539	168	Flagellar basal-body rod protein FlgB	Flagellum, Flagellum in Campylobacter	Flagellar motility in Prokaryota
66	NODE_8_len_273711_cov_22.2621_ID_15	32462	31644	819	Flagellin protein FlaA	Flagellum, Flagellum in Campylobacter	Flagellar motility in Prokaryota
67	NODE_8_len_273711_cov_22.2621_ID_15	33527	32721	807	Flagellin protein FlaA	Flagellum, Flagellum in Campylobacter	Flagellar motility in Prokaryota
68	NODE_8_len_273711_cov_22.2621_ID_15	53574	54380	807	Flagellin protein FlaA	Flagellum, Flagellum in Campylobacter	Flagellar motility in Prokaryota
69	NODE_8_len_273711_cov_22.2621_ID_15	82498	83322	825	Flagellin protein FlaA	Flagellum, Flagellum in Campylobacter	Flagellar motility in Prokaryota

Table S7: Chitin and N-acetyl glucosamine utilization genes in Bar D5

S.No.	Contig ID/ Length	Start codon	Stop codon	Length (bp)	Function	Subsystem	Sub category
1	NODE_2_len_608628_cov_33.798_ID_3	46927	45050	1878	PTS system, N-acetylglucosamine-specific IIB component (EC 2.7.1.69) / PTS system, N-acetylglucosamine-specific IIC component (EC 2.7.1.69)	Chitin and N-acetylglucosamine utilization,<br>Chitin and N-acetylglucosamine utilization,<br>Sialic Acid Metabolism,<br>Sialic Acid Metabolism	Aminosugars
2	NODE_2_len_608628_cov_33.798_ID_3	47142	47360	219	N-acetylglucosamine-6-phosphate deacetylase (EC 3.5.1.25)	Chitin and N-acetylglucosamine utilization,<br>Sialic Acid Metabolism	Aminosugars
3	NODE_2_len_608628_cov_33.798_ID_3	47452	48441	990	N-acetylglucosamine-6-phosphate deacetylase (EC 3.5.1.25)	Chitin and N-acetylglucosamine utilization,<br>Sialic Acid Metabolism	Aminosugars
4	NODE_2_len_608628_cov_33.798_ID_3	48444	49175	732	Glucosamine-6-phosphate deaminase (EC 3.5.99.6)	Chitin and N-acetylglucosamine utilization,<br>Sialic Acid Metabolism	Aminosugars
5	NODE_2_len_608628_cov_33.798_ID_3	49317	50042	726	Predicted transcriptional	Chitin and N-acetylglucosamine	Aminosugars

					regulator of N-Acetylglucosamine utilization, GntR family	utilization	
6	NODE_2_len_608628_cov_33.798_ID_3	202128	201235	894	N-acetylglucosamine kinase of eukaryotic type (EC 2.7.1.59)	Chitin and N-acetylglucosamine utilization	Aminosugars
7	NODE_2_len_608628_cov_33.798_ID_3	285439	284561	879	N-Acetyl-D-glucosamine ABC transport system, permease protein 2	Chitin and N-acetylglucosamine utilization	Aminosugars
8	NODE_6_len_357440_cov_16.0934_ID_11	262603	261950	654	Glucosamine-6-phosphate deaminase (EC 3.5.99.6)	Chitin and N-acetylglucosamine utilization,<br>Sialic Acid Metabolism	Aminosugars
9	NODE_9_len_231696_cov_18.7531_ID_17	79937	78831	1107	N-acetylglucosamine-6-phosphate deacetylase (EC 3.5.1.25)	Chitin and N-acetylglucosamine utilization,<br>Sialic Acid Metabolism	Aminosugars
10	NODE_2_len_608628_cov_33.798_ID_3	47142	47360	219	N-acetylglucosamine-6-phosphate deacetylase (EC 3.5.1.25)	Chitin and N-acetylglucosamine utilization,<br>Sialic Acid Metabolism	Aminosugars

Table S8: Top twenty closest neighbouring strains of *Bacillus* sp. strain D5 based on RAST annotation

Genome ID	Score	Genome name
545693.3	546	*Bacillus megaterium* QM B1551
592022.4	488	*Bacillus megaterium* DSM319
491915.4	357	*Anoxybacillus flavithermus* WK1
491915.6	349	*Anoxybacillus flavithermus* WK1
581103.3	233	*Geobacillus* sp. Y4.1MC1
471223.3	231	*Geobacillus* sp. WCH70
634956.3	229	*Geobacillus thermoglucosidasius* C56-YS93
581103.5	227	*Geobacillus* sp. Y4.1MC1
313627.6	221	*Bacillus* sp. NRRL B-14911
315749.4	204	*Bacillus cereus* subsp. cytotoxis NVH 391-98
315749.8	199	*Bacillus cytotoxicus* NVH 391-98
279010.12	180	*Bacillus licheniformis* ATCC 14580
279010.13	174	*Bacillus licheniformis* ATCC 14580
161544.4	173	*Bacillus* sp. SG-1
279010.5	168	*Bacillus licheniformis* ATCC 14580
665958.3	167	*Bacillus* sp. BT1B_CT2
334727.3	158	*Bacillus atrophaeus* C89
720555.4	147	*Bacillus atrophaeus* 1942
720556.3	141	*Bacillus atrophaeus* Detrick-1
665959.3	139	*Bacillus* sp. 2_A_57_CT2
